# Association between physical activity and thyroid function in American adults: a survey from the NHANES database

**DOI:** 10.1186/s12889-024-18768-4

**Published:** 2024-05-10

**Authors:** Lijun Tian, Cihang Lu, Weiping Teng

**Affiliations:** 1 Department of Endocrinology and Metabolism, The First Hospital of China Medical University, No. 155, Nanjing Bei Street, Shenyang, People’s Republic of China; 2 Institute of endocrinology, The First Hospital of China Medical University, Shenyang, China

**Keywords:** Physical activity, Thyroid function, NHANES, American adults, TSH

## Abstract

**Objective:**

Physical activity (PA) is closely related to our lives, and the effects of PA on thyroid function have not been elucidated.

**Methods:**

Using data from the National Health and Nutrition Examination Survey (NHANES) 2007–2012, we included 5877 participants and analyzed the associations of thyroid function with weekly physical activity (PAM, expressed in metabolic equivalents of task) and physical activity time (PAT) in American adults. Univariate and multivariate logistic analyses were used to demonstrate the associations of PAM and PAT with the primary outcome. Linear regression analysis was performed to determine the associations between thyroid biochemical indicators/diseases and PAM/PAT.

**Results:**

Our study revealed noticeable sex differences in daily PA among the participants. The odds ratio of the fourth versus the first quartile of PAM was 3.07 (confidence interval, CI [1.24, 7.58], *p* = 0.02) for overt hypothyroidism, 3.25 (CI [1.12, 9.45], *p* = 0.03) for subclinical hyperthyroidism in adult men. PAT in the range of 633–1520 min/week was found to be associated with the occurrence of subclinical hyperthyroidism [*p* < 0.001, OR (95% CI) = 5.89 (1.85, 18.80)], PAT of the range of > 1520 min/week was found to be associated with the occurrence of overt hypothyroidism [*p* < 0.001, OR (95% CI) = 8.70 (2.80, 27.07)] and autoimmune thyroiditis (AIT) [*p* = 0.03, OR (95% CI) = 1.42 (1.03, 1.97)] in adult men. When PAM < 5000 MET*minutes/week or PAT < 1000 min/week, RCS showed an L-shaped curve for TSH and an inverted U-shaped curve for FT4. The changes in FT3 and TT3 in men were linearly positively correlated with PAM and PAT, while TT4 is linearly negatively correlated.

**Conclusion:**

The amount of daily physical activity of American adults is strongly associated with changes in thyroid function, including thyroid hormone levels and thyroid diseases. Thyroid hormone levels were varied to a certain extent with changes in PAM and PAT.

**Supplementary Information:**

The online version contains supplementary material available at 10.1186/s12889-024-18768-4.

## Introduction

The thyroid and the thyroid hormones (THs) have physiological effects on various body targets, which are crucial for human energy metabolism [1, 2]. Thyroid dysfunction is very common in clinical practice, and iodine is the key factor in thyroid dysfunction [3, 4]. Hyperthyroidism (including clinical and subclinical forms) and hypothyroidism (including clinical and subclinical forms) are the two basic types of thyroid dysfunctions [5]. In the National Health and Nutrition Examination Survey (NHANES) III, the prevalence of hypothyroidism was 4.6% (0.3% clinical and 4.3% subclinical), and the prevalence of hyperthyroidism was 1.3% (0.5% clinical and 0.7% subclinical) [6]. At present, thyroid laboratory indexes (TSH, FT4, TT4, FT3, TT3, TPOAb, TgAb, etc.) are the primary methods used to evaluate thyroid function, thus providing a basis for disease diagnosis.

Caspersen proposed a definition for physical activity (PA) in 1985, namely, any physical movement produced by skeletal muscle that leads to energy consumption [7]. The PA we carry out in our daily lives mainly includes work and physical exercise. A certain degree of PA can promote growth and development, increase feelings of happiness, and improve sleep [8]. Tucker reported that compared with sedentary people, adults who participated in high levels of PA tended to have longer telomeres, which can significantly delay cell aging [9]. Studies have shown that a large proportion of deaths among adults older than 40 years of age in the United States can be attributed to insufficient levels of PA, and 110,000 deaths could be prevented annually if individuals aged 40 to 85 or older increased their MVPA (moderate-to-vigorous-intensity physical activity) by 10 min per day among US adults [10, 11]. Xu found that PA is an important protective factor for diabetes patients [12]. PA can also impact cardiovascular function, and prolonged exercise is associated with an increase in cardiac troponin levels [13, 14]. In addition, Byeon's survey on Korean elderly people who were living alone revealed that sustained flexibility exercise is effective in maintaining a healthy mental state [15].

At present, there are few studies on the relationship between thyroid function and PA. A recent cohort study showed no association between thyroid function, as defined by TSH and FT4, and total PA [16]. Given the unclear relationship between thyroid dysfunction and PA, we aimed to better explore the possible changes in thyroid function caused by different levels of PA and thereby inform people of the correct amount of exercise.

## Method

### Study design

**Fig. 1:**
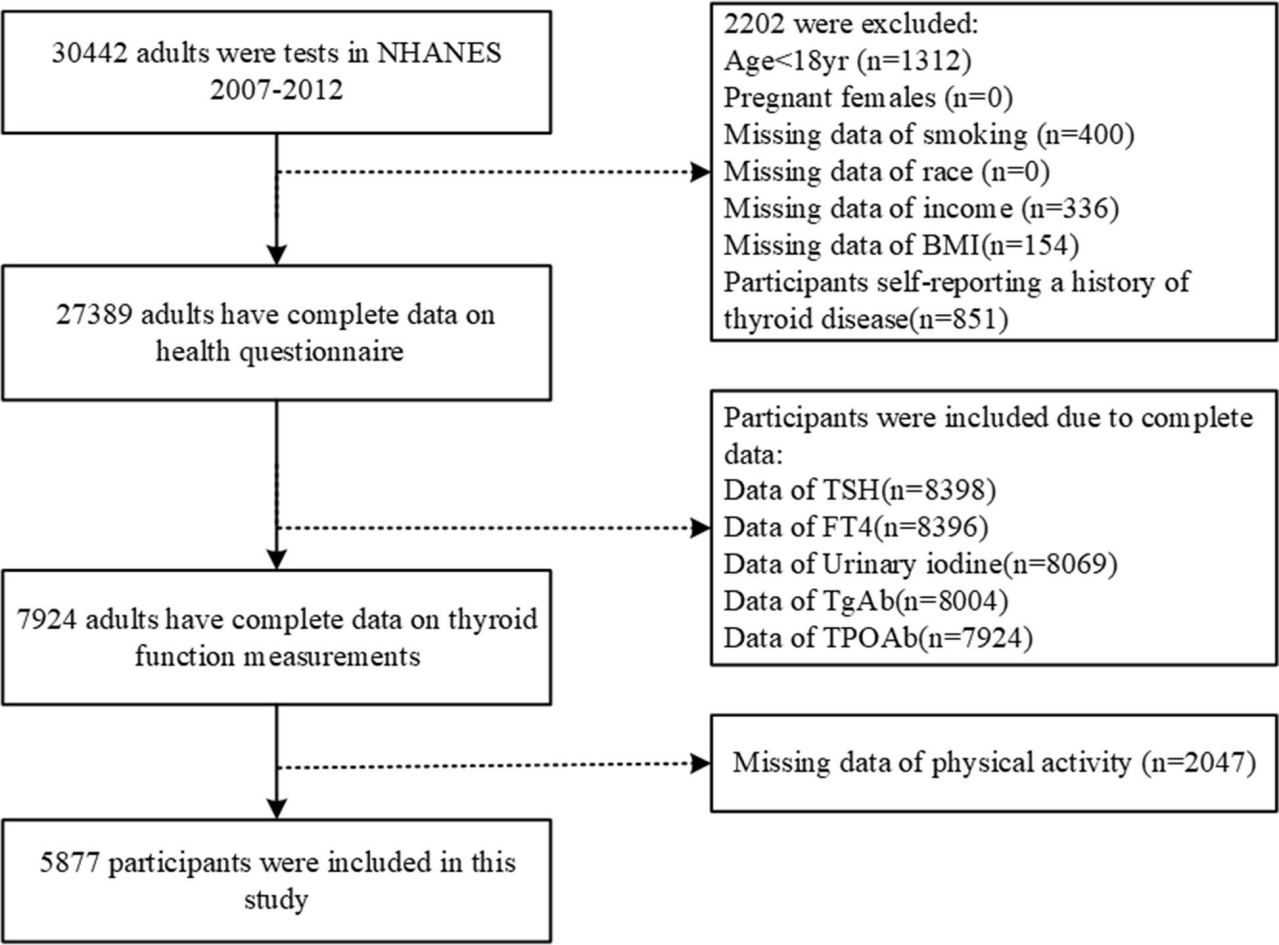
Flowchart of participant selection in the study, NHANES 2007–2012. The dotted lines represent participant exclusion

In this cross-sectional study, after excluding pregnant women and ambiguous or missing data, we included a total of 5,877 participants who were aged between 18 and 85 years and self-reported no history of thyroid disease for statistical analysis (Fig. 1). Exclusion criteria: age less than 18 years, lack of complete questionnaire information, self-reported history of thyroid disease. Inclusion criteria: complete data on thyroid laboratory tests, complete data on physical activity. Data were taken from the NHANES (NHANES—National Health and Nutrition Examination Survey Homepage (cdc.gov)) between 2007 and 2012. The NHANES database provided information on participants' work and PA, and activity-related variables were obtained through specific questionnaires. NHANES is a National Center for Health Statistics (NCHS) research program designed to assess the health and nutritional status of adults and children in the United States.

### Covariates

The covariates measured through the questionnaire mainly included age, sex (male or female), BMI, race (Mexican American, Non-Hispanic Black, Non-Hispanic White, Other Hispanic, and Other Races, including multiracial), self-reported education level (above and below university education), income (greater than $20,000, less than $20,000), smoking (never, former and current), and thyroid dysfunction (yes or no). The questionnaire was administered to people who did not report thyroid dysfunction.

### Clinical characteristics

Participants were measured for TSH, FT4, TT4, FT3, TT3, TPOAb, and TgAb to determine thyroid function. Various thyroid disorders, including overt hyperthyroidism, subclinical hyperthyroidism, overt hypothyroidism, subclinical hypothyroidism and autoimmune thyroiditis, were considered. The range of each chemical index and the diagnostic criteria of the disease were summarized as follows: TSH (0.34–5.6 mIU/L), FT4 (7.74–20.6 pmol/L), TT4 (4.5–13.2 µg/dL), FT3 (2.5–3.9 pg/mL), TT3 (87–178 ng/dL), TPOAb (< 34 IU/mL), TgAb (< 4. 0 IU/mL). Subclinical hyperthyroidism was defined as TSH < 0.34 mIU/L, FT3 and FT4 within the normal range. Overt hyperthyroidism was defined as TSH < 0.34 mIU/L, FT4 > 20.6 pmol/L. Subclinical hypothyroidism was defined as TSH > 5.6 mIU/L, FT4 within normal range. Overt hypothyroidism was defined as TSH > 5.6 mIU/L, fT4 < 7.74 pmol/L. Autoimmune thyroiditis (AIT) was defined as increased serum concentrations of thyroid antibodies [antithyroid peroxidase (microsomal) (TPOAb) and antithyroglobulin (TgAb)] [2, 17–19].

### Physical activity

Exercise intensity is usually expressed by the metabolic equivalent of task (MET), which refers to the ratio of energy consumed during PA to energy consumed at rest [20, 21]. One MET is equivalent to the resting metabolic rate or energy expenditure when awake and sitting quietly. Moderate-intensity PA has a MET value of 3 to 5.9 MET; vigorous PA has a MET value of 6 or higher [8]. Total population PA was analyzed according to the recommended MET scores, including active work-related activity (high-intensity, 8.0), moderate work-related activity (moderate-intensity, 4.0), walking or biking for transportation (4.0), vigorous recreational PA (8.0), and moderate leisure-time PA (4.0) (PAQ_E (cdc.gov)). Participants self-reported based on questionnaire items, and the questionnaire was developed by the National Center for Health Statistics (NCHS). MET score * the time spent on activities corresponding to a week was defined as the total physical activity MET (PAM) of the week, expressed in MET-min/week; physical activity time (PAT) was defined as the total activity time in a week, expressed in min/week.

### Statistical analysis

We conducted complex sampling of NHANES data from three survey cycles (2007–2008, 2009–2010, and 2011–2012) using R version 4.2.3. To ensure that our prevalence estimates were representative of the US population, we applied the MEC weights. Data were first analyzed for normality of distribution using the Kolmogorov‒Smirnov test of normality. Continuous variables are expressed as the mean and 95% confidence intervals (mean, 95%CI), and categorical variables are expressed as the rate and 95% confidence intervals (%, 95%CI). Different populations were divided into four groups according to their respective PAM and PAT quartiles, and intergroup differences were compared using the Mann‒Whitney U test. As an example, for male participants, PAM, measured in MET-minutes/week, was divided into four quartiles: Q1 < 1200, Q2 1200–3320, Q3 3320–8400, Q4 > 8400 MET-minutes/week, and PAT, measured in minutes/week, was also divided into four quartiles: Q1 (< 240), Q2 (240–633), Q3 (633–1520), and Q4 (> 1520). Differences in baseline characteristics across PAM and PAT quartiles were compared using the Kruskal‒Wallis test. Differences among groups were compared through the chi-square test or Fisher’s exact test. We conducted a binary logistic regression analysis to investigate the association between PAM, PAT and thyroid function and visualized the results using restricted cubic splines to illustrate the correlation between PA, thyroid biochemical markers, and thyroid function disorders. We also conducted multivariable regression analysis after adjusted for race/ethnicity, income, smoking status, multiple tests of PA and thyroid function disorders was corrected by the Tukey–Kramer corrections. The odds ratios (ORs) are presented with 95% confidence intervals (CIs), and *P* values less than 0.05 were considered indicative of statistical significance in all tests.

## Results

We included 5877 participants in the NHANES from 2007 to 2012 (mean age = 44.23 years, range 18–85 years), of which 3188 were males (53.05%) and 2759 were females (46.95%). The clinical characteristics of the participants are shown in Table 1. In the total population, the antibody positivity rates for TPOAb and TgAb were significantly higher in women than in men (15.37% > 7.69%, *p* < 0.001; 9.83% > 5.50%, *p* < 0.001). The prevalence of subclinical hypothyroidism was higher in women than in men (2.6% > 1.57%, *p* = 0.01). In the subgroup of TPOAb greater than 34 IU/mL and TgAb greater than 4 IU/mL, the proportion of females was much higher than that of males. Women are more likely to have thyroid dysfunction than men (7.24% > 4.96%, *p* = 0.01).

**Table 1:** Baseline characteristics of American adults aged 18–85 years stratified by sex, NHANES 2007–2012

Variable	Total	Male	Female	*P* value
N (%)	5877	3118(53.05%)	2759(46.95%)	
Weighted N	51989539	42238675(51.52%)	39750864(48.48%)	
Age (yr)^a^	44.23(43.41,45.05)	43.68(42.80,44.56)	44.81(43.81,45.81)	0.02
BMI^a^	28.02(27.77,28.27)	28.25(27.73,28.31)	27.77(27.46,28.08)	0.01
**Social and economic parameters**				
Race (%)^a^				0.01
Non-Hispanic White	71.65(62.44,80.87)	70.12(65.46,74.77)	73.30(69.36,77.24)	
Non-Hispanic Black	9.52(7.63,11.42)	9.46(7.33,11.59)	9.59(7.32,11.87)	
Mexican American	7.66(6.25, 9.06)	8.86(7.07,10.65)	6.37(4.76, 7.98)	
Other Hispanic	4.78(3.53, 6.04)	4.92(3.40,6.44)	4.63(3.38,5.88)	
Other Races – Including Multi-racial	6.38(5.09, 7.67)	6.64(4.77,8.52)	6.11(4.75,7.46)	
Education (%)^a^				< 0.001
Lower University	37.14(32.79,41.49)	41.81(37.77,45.85)	35.39(32.14,38.65)	
Higher University	58.83(52.88,64.78)	58.19(54.15,62.23)	64.61(61.35,67.86)	
Income ($)^a^				0.002
< 20,000	16.62(14.44,18.79)	15.48(13.08,17.88)	18.93(16.23,21.63)	
> 20,000	80.29(72.47,88.11)	84.52(82.12,86.92)	81.07(78.37,83.77)	
Smoke (%)^a^				< 0.001
Never	52.14(47.31,56.97)	47.94(45.34,50.53)	61.09(58.71,63.46)	
Former	23.58(21.31,25.85)	28.44(26.15,30.73)	20.44(18.48,22.39)	
Current	20.29(17.95,22.62)	23.62(21.39,25.86)	18.48(16.32,20.64)	
**Thyroid parameters**				
TSH (mIU/L)^a^	2.04(1.94,2.14)	1.93(1.81,2.05)	2.15(1.99,2.31)	0.04
FT4(pmol/L)^a^	10.24(10.10,10.38)	10.23(10.09,10.37)	10.26(10.12,10.40)	0.65
FT3(pg/mL)^a^	3.20(3.18,3.22)	3.30(3.28,3.32)	3.09(3.07,3.11)	< 0.001
TT4(ug/dL)^a^	7.77(7.69,7.85)	7.55(7.47,7.63)	8.01(7.91,8.11)	< 0.001
TT3(ng/dl)^a^	114.27(112.98,115.56)	115.73(114.28,117.18)	112.71(111.42,114.28)	< 0.001
TPOAb(IU/mL)^a^	21.78(19.58,23.98)	13.97(11.09,16.85)	30.20(25.93,34.47)	< 0.001
TgAb(IU/mL)^a^	10.00(7.30,12.70)	7.77(4.07,11.47)	12.39(8.29,16.49)	0.11
Thyroid diseases (%)^a^				0.004
Subclinical hypothyroidism	2.07(1.52, 2.61)	1.57(1.00,2.14)	2.60(1.81,3.40)	0.01
Subclinical hyperthyroidism	1.50(1.09, 1.91)	0.90(0.53,1.28)	2.15(1.45,2.84)	0.07
Overt hypothyroidism	2.24(1.69, 2.80)	2.32(1.57,3.08)	2.16(1.59,2.73)	0.77
Overt hyperthyroidism	0.25(0.09, 0.40)	0.16(0,0.35)	0.34(0.09,0.58)	0.37
PTPOAb (IU/mL)^a,b^				< 0.001
< 34	87.92(80.72,95.13)	92.31(90.85,93.77)	84.63(82.97,86.30)	
> 34	11.29(9.78,12.81)	7.69(6.23, 9.15)	15.37(13.70,17.03)	
PTgAb (IU/mL)^a,b^				< 0.001
< 4	91.88(84.37,99.39)	94.50(93.32,95.68)	90.17(88.61,91.73)	
> 4	7.55(6.46, 8.63)	5.50(4.32, 6.68)	9.83(8.27,11.39)	
Thyroid Dysfunction (%)^a^				0.01
No	93.94(86.46,101.42)	95.04(94.06,96.03)	92.76(91.46,94.05)	
Yes	6.06(4.98, 7.14)	4.96(3.97,5.94)	7.24(5.95,8.54)	

*Abbreviations*: *BMI* Body mass index, *TSH* Thyroid-stimulating hormone or thyrotropin, *FT3* Free triiodothyronine, *FT4* Free thyroxine, *TgAb* Thyroglobulin antibody, *TPOAb* Thyroid peroxidase antibody

^a^presented as mean (95% confidence interval)

^b^PTPOAb means the division by the TPOAb positive range (34 IU/mL); PTgAb means the division by the TPOAb positive range (4 IU/mL)

The adjusted ORs for PA among the total population and men are shown in Table 2, which lists the results of the univariate (Model 1), age-adjusted, BMI-adjusted (Model 2), and multivariate (Model 3) logistic regressions for thyroid function and PA in men. Our findings showed that in Model 1, a longer PAT was associated with subclinical hypothyroidism in men [*p* = 0.04, OR (95% CI) = 0.17 (0.03, 0.91)]. In both Model 2 and Model 3, PAT in Q4 was shown to be associated with the overt hypothyroidism in the total population [*p* = 0.02, OR (95% CI) = 2.41 (1.17,4.96); *p* = 0.01, OR (95% CI) = 2.72 (1.130, 5.71)]. In Model 3, the odds ratio of the second versus the first quartile of PAT was [*p* = 0.02, OR (95% CI) = 23.35 (1.89, 288.23)] for hyperthyroidism, and PAT in men was significantly associated with subclinical hyperthyroidism, overt hypothyroidism and AIT [*p* < 0.001; OR (95% CI) = 5.89 (1.85, 18.8); *p* < 0.001, OR (95% CI) = 8.7 (2.80, 27.07); *p* = 0.03, OR (95% CI) = 1.42 (1.03,1.97)]. Regarding PAM, we did not observe a significant association between PAM and thyroid diseases in the total population, but in men, high PAM was associated with subclinical hypothyroidism in Model 1 [*p* = 0.04, OR (95% CI) = 0.22 (0.05, 0.94)], and Model 3 showed that high PAM was correlated with subclinical hyperthyroidism and overt hypothyroidism [*p* = 0.03, OR (95% CI) = 3.25 (1.12, 9.45)] [*p* = 0.02, OR (95% CI) = 3.07 (1.24, 7.58)].

**Table 2:** Multivariate-adjusted association of physical activity and thyroid dysfunction in US adults 18–85 years, NHANES 2007–2012

**PAT**			SHYPER P OR (95% CI)		OHYPER P OR (95% CI)		SHYPO P OR (95% CI)		OHYPO P OR (95% CI)		AIT P OR (95% CI)	
		Q1	ref	ref	ref	ref	ref	ref	ref	ref	ref	ref
	Model1	Q2	0.42	0.74(0.35,1.57)	0.74	0.90(0.46,1.74)	0.23	1.51(0.76,3.00)	0.2	1.50(0.80,2.82)	0.74	0.95(0.68,1.32)
		Q3	0.95	0.97(0.44,2.17)	0.84	0.93(0.45,1.93)	0.81	1.09(0.53,2.23)	0.94	1.04(0.42,2.58)	0.21	0.80(0.56,1.14)
		Q4	0.11	0.51(0.23,1.16)	0.07	0.48(0.21,1.06)	0.25	0.62(0.28,1.41)	0.07	1.92(0.96,3.86)	0.25	0.84(0.62,1.13)
		Q1	ref	ref	ref	ref	ref	ref	ref	ref	ref	ref
	Model2	Q2	0.34	0.73(0.37,1.41)	0.49	1.64(0.39,6.80)	0.52	1.25(0.62,2.54)	0.17	1.67(0.80,3.48)	0.98	0.99(0.67,1.49)
**Total**		Q3	0.92	0.96(0.41,2.25)	0.55	0.61(0.12,3.16)	0.94	1.03(0.49,2.18)	0.71	1.21(0.44,3.29)	0.87	1.04(0.68,1.59)
		Q4	0.66	0.85(0.39,1.84)	0.09	0.22(0.04,1.30)	0.55	0.79(0.36,1.75)	**0.02**	**2.41(1.17, 4.96)**	0.66	1.09(0.73,1.64)
		Q1	ref	ref	ref	ref	ref	ref	ref	ref	ref	ref
	Modle3	Q2	0.36	0.73(0.37,1.46)	0.46	1.70(0.40, 7.19)	0.58	1.22(0.59,2.53)	0.08	1.87(0.93,3.77)	0.88	1.03(0.73,1.43)
		Q3	0.89	0.94(0.39,2.26)	0.59	0.65(0.12, 3.37)	1	1.00(0.47,2.12)	0.55	1.35(0.49,3.70)	0.75	0.94(0.62,1.41)
		Q4	0.71	0.86(0.39,1.91)	0.09	0.21(0.03, 1.30)	0.63	0.83(0.37,1.83)	**0.01**	**2.72(1.30,5.71)**	0.17	1.25(0.90,1.74)
		Q1	ref	ref	ref	ref	ref	ref	ref	ref	ref	ref
	Modle1	Q2	0.14	2.55(0.73, 8.93)	**0.02**	**3.86(1.21,12.38)**	0.49	1.49(0.48,4.66)	0.73	1.15(0.52,2.51)	0.9	0.97(0.62,1.52)
		Q3	**0.01**	**5.04(1.56,16.34)**	**0.01**	**4.57(1.48,14.10)**	0.18	1.67(0.78,3.57)	0.12	2.16(0.81,5.76)	0.17	0.67(0.38,1.19)
		Q4	0.28	1.93(0.58, 6.42)	0.29	1.90(0.57, 6.31)	**0.04**	**0.17(0.03,0.91)**	**0.01**	**3.16(1.43,6.99)**	0.41	1.15(0.82,1.63)
		Q1	ref	ref	ref	ref	ref	ref	ref	ref	ref	ref
**Male**	Model2	Q2	0.13	2.58(0.75, 8.91)	**0.01**	**21.85(1.93,247.07)**	0.57	1.41(0.42,4.72)	0.94	1.03(0.43,2.50)	0.98	0.99(0.64,1.55)
		Q3	**0.01**	**5.56(1.72,17.95)**	**-**	**-**	0.12	1.83(0.86,3.90)	0.07	2.49(0.92,6.78)	0.27	0.73(0.41,1.29)
		Q4	0.18	2.19(0.68, 7.07)	0.59	2.29(0.11, 48.38)	0.07	0.20(0.03,1.17)	**0.01**	**3.75(1.42,9.87)**	0.12	1.31(0.93,1.85)
		Q1	ref	ref	ref	ref	ref	ref	ref	ref	ref	ref
	Model3	Q2	0.12	2.63(0.77, 9.06)	**0.02**	**23.35(1.89,288.23)**	0.69	1.25(0.40,3.87)	**0.05**	**2.94(1.01, 8.52)**	0.82	0.95(0.61,1.49)
		Q3	** < 0.001**	**5.89(1.85,18.80)**	**-**	**-**	0.66	0.83(0.36,1.94)	**0.002**	**6.10(2.00,18.61)**	0.3	0.74(0.42,1.32)
		Q4	0.17	2.27(0.70, 7.35)	0.58	2.38(0.10, 55.51)	0.68	0.82(0.31,2.19)	** < 0.001**	**8.70(2.80,27.07)**	**0.03**	**1.42(1.03,1.97)**
**PAM**												
		Q1	ref	ref	ref	ref	ref	ref	ref	ref	ref	ref
	Model1	Q2	0.38	0.72(0.35,1.51)	0.66	0.87(0.46,1.65)	0.35	1.38(0.70,2.75)	0.35	1.29(0.75,2.21)	0.97	1.01(0.73,1.38)
		Q3	0.58	0.80(0.36,1.78)	0.47	0.77(0.37,1.60)	0.14	1.57(0.86,2.87)	0.95	0.97(0.43,2.21)	0.28	0.82(0.57,1.18)
		Q4	0.35	0.68(0.30,1.54)	0.21	0.61(0.28,1.34)	0.19	0.53(0.20,1.38)	0.15	1.55(0.84,2.84)	0.23	0.84(0.63,1.12)
		Q1	ref	ref	ref	ref	ref	ref	ref	ref	ref	ref
	Model2	Q2	0.48	0.77(0.36,1.62)	0.29	2.06(0.53,7.98)	0.21	1.52(0.78,2.95)	0.36	1.34(0.70,2.58)	0.43	1.14(0.82,1.57)
**Total**		Q3	0.95	0.97(0.42,2.25)	0.61	0.68(0.14,3.16)	0.06	1.79(0.98,3.29)	0.77	1.13(0.49,2.61)	0.86	1.03(0.70,1.52)
		Q4	0.95	1.03(0.46,2.27)	0.13	0.26(0.04,1.48)	0.48	0.71(0.27,1.87)	0.07	1.80(0.95,3.42)	0.11	1.26(0.95,1.68)
		Q1	ref	Ref	ref	ref	ref	ref	ref	ref	ref	ref
	Model3	Q2	0.55	0.79(0.36,1.73)	0.23	2.27(0.58, 8.89)	0.25	1.45(0.76,2.80)	0.28	1.44(0.73,2.85)	0.63	1.08(0.78,1.50)
		Q3	0.93	0.96(0.42,2.23)	0.64	0.70(0.15, 3.29)	0.08	1.73(0.94,3.19)	0.66	1.21(0.51,2.91)	0.97	1.01(0.68,1.50)
		Q4	0.88	1.06(0.48,2.35)	0.12	0.25(0.04, 1.43)	0.47	0.71(0.27,1.84)	0.06	1.96(0.97,3.94)	0.1	1.29(0.95,1.74)
		Q1	ref	ref	ref	ref	ref	ref	ref	ref	ref	ref
	Model1	Q2	0.19	2.21(0.67, 7.33)	0.08	2.36(0.91,6.13)	0.31	1.58(0.65,3.85)	0.09	0.44(0.17,1.13)	0.66	0.91(0.58,1.41)
		Q3	0.06	3.25(0.97,10.92)	0.16	2.29(0.71,7.37)	0.24	1.38(0.81,2.36)	0.23	1.81(0.68,4.81)	0.1	0.65(0.39,1.08)
		Q4	0.09	2.43(0.86, 6.91)	0.25	1.82(0.65,5.08)	**0.04**	**0.22(0.05,0.94)**	0.1	1.81(0.89,3.70)	0.99	1.00(0.70,1.44)
		Q1	ref	ref	ref	ref	ref	ref	ref	ref	ref	ref
	Model2	Q2	0.15	2.35(0.73, 7.56)	0.32	3.20(0.31,33.00)	0.36	1.52(0.60,3.85)	0.25	0.59(0.24,1.47)	0.78	0.94(0.60,1.47)
		Q3	**0.04**	**3.97(1.10,14.29)**	**-**	**-**	0.16	1.53(0.84,2.81)	**0.04**	**2.61(1.03,6.61)**	0.22	0.73(0.44,1.21)
**Male**		Q4	**0.04**	**2.95(1.06,8.20)**	0.55	0.45(0.03,6.57)	0.11	0.26(0.05,1.35)	**0.03**	**2.70(1.13,6.48)**	0.39	1.18(0.81,1.72)
		Q1	ref	ref	ref	ref	ref	ref	ref	ref	ref	ref
	Model3	Q2	0.14	2.39(0.75, 7.59)	0.34	3.12(0.29,33.48)	0.4	1.45(0.60,3.53)	0.43	0.70(0.28,1.75)	0.57	0.88(0.55,1.39)
		Q3	**0.03**	**4.09(1.16,14.51)**	**-**	**-**	0.13	1.58(0.87,2.85)	**0.02**	**3.07(1.26,7.49)**	0.21	0.72(0.43,1.22)
		Q4	**0.03**	**3.25(1.12,9.45)**	0.55	0.44(0.03, 6.56)	0.16	0.30(0.06,1.63)	**0.02**	**3.07(1.24,7.58)**	0.18	1.28(0.88,1.87)

The numbers in bold are statistically significant. ORs are estimated with logistic models

Model 1: bivariate model, include only physical activity (including PAM and PAT) and thyroid diseases. Model 2: multivariable models were adjusted for age, body mass index, gender (in overall sample). Model 3: additionally adjusted for race/ethnicity, income, smoking status

*Abbreviations*: *PAT* Physical activity metabolic equivalent, *PAT* Physical activity time, *SHYPER* Subclinical hyperthyroidism, *OHYPER* Overt hyperthyroidism, *SHYPO* Subclinical hypothyroidism, *OHYPO* Overt hypothyroidism, *AIT* Autoimmune thyroiditis, *Ref* Reference, *OR* Odds ratio, *CI* Confidence interval

We found that PAM and PAT were not substantially associated with thyroid function in women (Supplementary materials Table S1), so we mainly explored PA and thyroid function in the total population and in men in the subsequent analysis. Based on the quartiles of weekly PA recommended by the US physical activity guidelines, we divided the total population and men into four groups each to further compare the association between thyroid function and PA. In the total population, the higher the weekly PAM was, the greater the proportion of men (*p* < 0.0001) and percentage of current smokers (*p* < 0.0001). With increasing PAM, TSH, FT4, and TT4 levels showed an overall decreasing trend (*p* = 0.002; *p* < 0.001; *p* < 0.0001), while FT3 and TT3 levels gradually increased (*p* < 0.0001; *p* < 0.0001) and TPOAb showed a downward trend (p for trend = 0.008) (Supplementary materials Table S2).

**Fig. 2:**
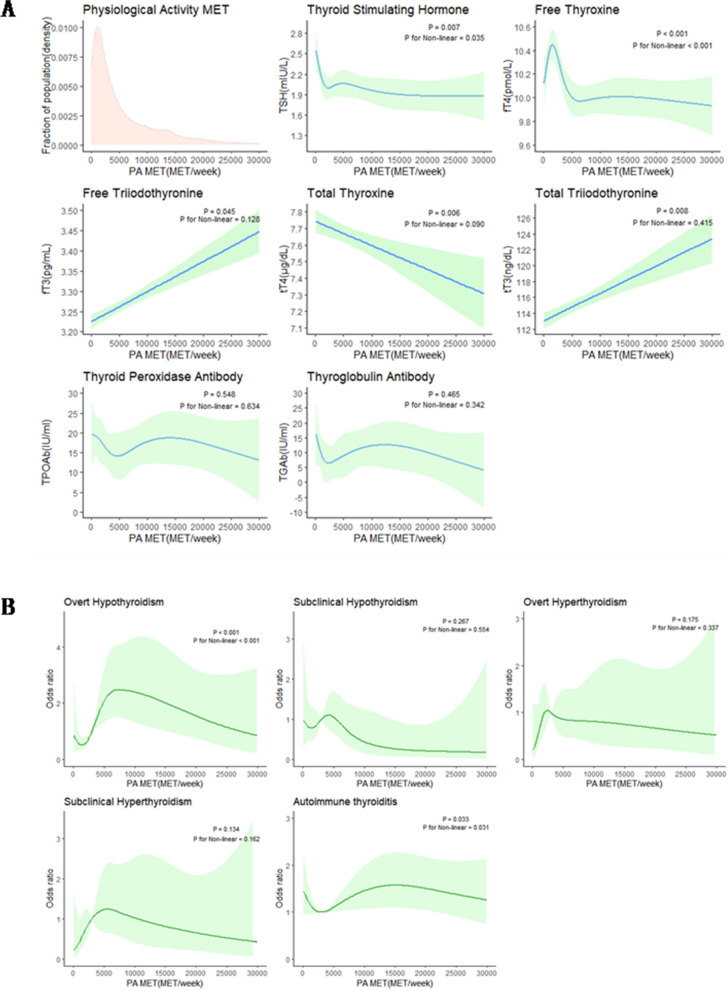
The relationship between the risk of thyroid dysfunction and the PAM during a week in men (restricted cubic spline fitting logistic regression, RCS). **A** Thyroid biochemical indicators; **B** Thyroid disorders. The light red shade is the population analysis density (density) on the x-axis, indicating where the population is mainly concentrated Side, the blue line refers to the fitting curve of the OR value as the x-axis index increases, and the green shade is the 96% confidence interval. The *P* value represents whether the trend of the x-axis and the y-axis is statistically significant. If the *P* value is less than 0.05, there is a trend relationship between X and Y as shown in the figure. When P is less than 0.1, it can also be said that the relationship between the two is not significant enough. The value of *P* for Nonlinear judges whether it is nonlinear. The value of *P* for Nonlinear is less than 0.05 to meet nonlinearity. The value of *P* for Nonlinear is greater than or equal to 0.05, indicating that XY is a linear relationship

In Table 3, the levels of TSH, FT4, and TT4 in males decreased significantly with increasing PAM (p for trend = 0.006; p for trend < 0.001; p for trend = 0.001), and FT3 and TT3 increased with increasing weekly PAM. To further analyze the relationship between thyroid and PA, we defined four groups according to the quartiles of TSH and FT4: the low TSH group (TSHL), the high TSH group (TSHH), the low FT4 group (FT4L) and the high FT4 group (FT4H). The TSHL group increased with increasing activity (p for trend = 0.014), while the FT4H group decreased with increasing activity (p for trend = 0.047). Then, we performed a logistic regression model was fitted with a restricted cubic spline (RCS) and found that within 5000 MET-minutes/week of PAM, TSH showed an “L” curve, and FT4 showed an “inverted U” curve; TT3 and FT3 were linearly positively correlated with PAM, while TT4 was linearly negatively correlated with PAM (Fig. 2A). Men were more likely to have thyroid dysfunction when the PAM was Q3, and in the RCS plots of thyroid disease and PAM, within 10,000 MET, overt hypothyroidism showed a J-shaped curve (*p* < 0.001), while AIT showed a U-shaped curve (*p* = 0.012) (Fig. 2B).

**Table 3:** Relationship between thyroid function and PAM in US men aged 18–85 years, NHANES 2007–2012

Variable	Total	< 1200MET-minutes/week	1200-3320MET-minutes/week	3320-8400MET-minutes/week	> 8400MET-minutes/week	*P* value	P for trend
**Age(yr)**	43.71(42.81,44.61)	48.65(47.32,49.98)	45.17(43.60,46.74)	41.97(40.54,43.40)	39.34(38.16,40.52)	< 0.001	
**BMI**	28.25(27.96,28.54)	28.82(28.09,29.55)	28.13(27.68,28.58)	28.24(27.87,28.61)	27.85(27.38,28.32)	< 0.001	
**Smoke**						< 0.001	
Never	61.33(55.07,67.59)	59.20(54.85,63.54)	66.30(62.14,70.45)	63.69(57.93,69.46)	56.25(51.61,60.90)		
Former	20.10(17.33,22.86)	23.14(19.42,26.85)	19.46(15.91,23.02)	20.35(16.04,24.66)	17.43(13.74,21.12)		
Current	18.57(15.84,21.30)	17.66(14.36,20.97)	14.24(10.82,17.66)	15.96(11.91,20.01)	26.32(22.81,29.83)		
**TSH (mIU/L)**	1.93(1.81,2.05)	2.16(1.75,2.57)	1.93(1.75,2.11)	1.92(1.80,2.04)	1.72(1.64,1.80)	0.01	0.006
**FT4(pmol/L)**	10.23(10.09,10.37)	10.33(10.15,10.51)	10.45(10.25,10.65)	10.11(9.95,10.27)	10.04(9.84,10.24)	< 0.01	< 0.001
**FT3(pg/mL)**	3.30(3.28,3.32)	3.25(3.23,3.27)	3.27(3.23,3.31)	3.33(3.29,3.37)	3.35(3.31,3.39)	< 0.001	< 0.001
**TT4(ug/dL)**	7.54(7.46,7.62)	7.72(7.60,7.84)	7.55(7.45,7.65)	7.48(7.34,7.62)	7.43(7.33,7.53)	0.01	0.001
**TT3(ng/dl)**	115.71(114.26,117.16)	113.91(111.03,115.79)	112.97(111.07,114.87)	117.45(114.90,120.00)	118.60(116.31,120.89)	< 0.001	< 0.001
**TPOAb (IU/mL)**^**a**^	13.96(11.10,16.82)	15.67(9.03,22.31)	15.72(10.21,21.23)	12.14(4.55,19.73)	12.30(6.46,18.14)	0.52	0.20
**TgAb (IU/mL)**	7.68(4.07,11.29)	11.35(2.49,20.21)	5.80(2.37, 9.23)	6.49(2.39,10.59)	7.53(0,15.06)	0.13	0.902
**TSHL (mIU/L)**						0.07	0.014
> 1.07	73.89(66.69,81.08)	75.37(71.19,79.56)	77.96(73.68,82.24)	71.20(66.69,75.71)	71.28(67.73,74.84)		
< 1.07	26.11(22.80,29.43)	24.63(20.44,28.81)	22.04(17.76,26.32)	28.80(24.29,33.31)	28.72(25.16,32.27)		
**TSHH (mIU/L)**						0.08	0.125
< 2.33	74.90(67.99,81.81)	71.82(67.93,75.71)	71.90(66.91,76.88)	78.97(74.66,83.28)	76.62(72.32,80.91)		
> 2.33	25.10(21.69,28.51)	28.18(24.29,32.07)	28.10(23.12,33.09)	21.03(16.72,25.34)	23.38(19.09,27.68)		
**FT4L(pmol/L)**						0.02	0.073
> 9	86.33(78.78,93.88)	88.75(86.37,91.13)	81.96(77.55,86.37)	88.48(84.98,91.97)	85.89(81.67,90.12)		
< 9	13.67(10.67,16.68)	11.25(8.87,13.63)	18.04(13.63,22.45)	11.52(8.03,15.02)	14.11(9.88,18.33)		
**FT4H (pmol/L)**						0.15	0.047
< 11.5	75.09(66.57,83.62)	71.67(66.49,76.85)	75.07(70.66,79.48)	74.80(70.66,78.94)	78.81(73.90,83.71)		
> 11.5	24.91(21.84,27.97)	28.33(23.15,33.51)	24.93(20.52,29.34)	25.20(21.06,29.34)	21.19(16.29,26.10)		
**Thyroid**						0.01	
Subclinical hypothyroidism	1.58(0.97, 2.19)	1.26(0.37,2.14)	1.94(0.68,3.20)	2.78(1.65,3.91)	0.27(0,0.62)	0.02	
Subclinical hyperthyroidism	0.90(0.51, 1.29)	0.60(0,1.21)	0.83(0.05,1.61)	1.22(0.22,2.22)	0.94(0.36,1.52)	0.02	
Overt hypothyroidism	2.32(1.52, 3.12)	2.02(1.02,3.02)	0.82(0.28,1.36)	3.28(1.21,5.36)	3.25(1.55,4.95)	0.02	
Overt hyperthyroidism	0.17(0, 0.34)	0.16(0,0.32)	0.42(0,1.09)	0.00(0.00,0.00)	0.06(0,0.12)	0.06	
AIT	18.23(15.52,20.94)	19.79(15.90,23.68)	18.21(14.42,22.00)	17.52(13.69,21.35)	18.66(15.00,22.33)		
**Thyroid Dysfunction**						0.05	
No	95.04(87.77,102.31)	95.97(94.27,97.66)	95.99(94.28,97.69)	92.71(90.69,94.74)	95.48(93.58,97.38)		
Yes	4.96(3.80, 6.12)	4.03(2.34,5.73)	4.01(2.31,5.72)	7.29(5.26,9.31)	4.52(2.62,6.42)		

All data was presented as mean (95% confidence interval). Participants had TSH quartiles of 1.57 (1.07–2.33) mIU/L and FT4 quartiles of 10.3 (9–11.5) pmol, TSHL, the low TSH quartile array; TSHH, the high TSH quartile array; FT4L, the low FT4 quartile array; FT4H, the high FT4 quartile array. *P*-Values are for differences between groups

**Fig. 3:**
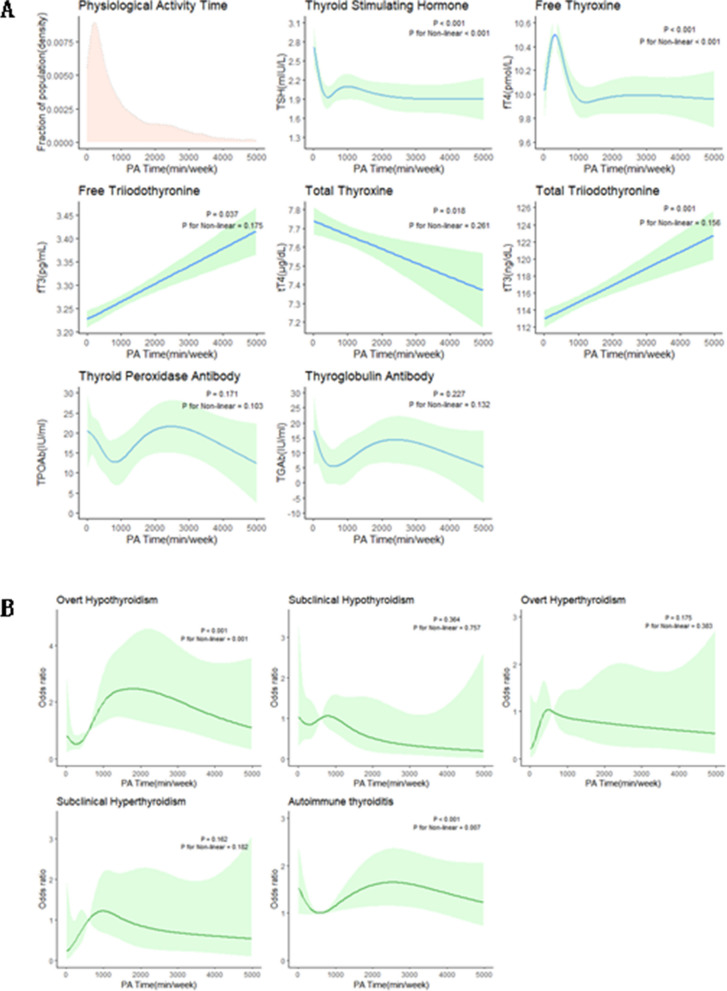
The relationship between the risk of thyroid dysfunction and the PAT during a week in men (restricted cubic spline fitting logistic regression, RCS). **A** Thyroid biochemical indicators; **B** Thyroid disorders

Then, we analyzed weekly PAT and found that TSH, FT4, TT4, FT3, TT3 and TPOAb levels changed in the total population PAT with the same trend as PAM. The TSHL group increased with increasing PAT (p for trend = 0.034), and the FT4H group decreased with increasing PAT (p for trend = 0.022) (Supplementary materials Table S3). In men, FT4 and TT4 were negatively correlated with PAT (p for trend < 0.001; p for trend = 0.008), and FT3 and TT3 were positively correlated with PAT (p for trend < 0.0001; p for trend < 0.0001). The FT4H group decreased with increasing PAT in men (p for trend = 0.013) (Table 4). The RCS plot showed that in men, when PAT was within 1000 min/week, the trends of TSH, FT4, TT3, FT3, and TT4 were the same as PAM (Fig. 3A) and the same trend in the prevalence risk of overt hypothyroidism and AIT as for PAM was observed (Fig. 3B). Considering that antibodies may introduce certain confounding influences on the results, we conducted further analysis on 5,167 participants after excluding 710 participants who tested positive for antibodies (Supplementary materials Table S4, S5). After reanalysis, there were no obvious changes observed in the results.

**Table 4:** Relationship between thyroid function and PAT in US men aged 18–85 years, NHANES 2007–2012

Variable	Total	< 240 minutes/week	240–633 minutes/week	633–1520 minutes/week	> 1520 minutes/week	*P* value	P for trend
**Age (yr)**	43.71(42.81,44.61)	46.80(45.39,48.21)	46.26(45.10,47.42)	43.48(41.64,45.32)	40.38(39.11,41.65)	< 0.001	
**BMI**	28.25(27.96,28.54)	29.03(28.30,29.76)	28.18(27.77,28.60)	28.01(27.62,28.40)	28.11(27.70,28.52)	0.05	
**Smoke**						< 0.001	
Never	61.33(55.07,67.59)	59.20(54.85,63.54)	66.30(62.14,70.45)	63.69(57.93,69.46)	56.25(51.61,60.90)		
Former	20.10(17.33,22.86)	23.14(19.42,26.85)	19.46(15.91,23.02)	20.35(16.04,24.66)	17.43(13.74,21.12)		
Current	18.57(15.84,21.30)	17.66(14.36,20.97)	14.24(10.82,17.66)	15.96(11.91,20.01)	26.32(22.81,29.83)		
**TSH (mIU/L)**	1.93(1.81,2.05)	2.24(1.65,2.83)	1.93(1.75,2.11)	1.93(1.81,2.05)	1.78(1.68,1.88)	0.002	0.023
**FT4(pmol/L)**	10.23(10.09,10.37)	10.29(10.07,10.51)	10.46(10.28,10.64)	10.26(10.06,10.46)	10.00(9.82,10.18)	< 0.001	< 0.001
**FT3(pg/mL)**	3.30(3.28,3.32)	3.27(3.23,3.31)	3.25(3.21,3.29)	3.30(3.26,3.34)	3.35(3.31,3.39)	< 0.001	< 0.001
**TT4(ug/dL)**	7.54(7.46,7.62)	7.68(7.50,7.86)	7.62(7.50,7.74)	7.52(7.36,7.68)	7.43(7.29,7.53)	0.01	0.008
**TT3(ng/dl)**	115.71(114.26,117.16)	114.13(112.13,116.13)	112.84(111.0,114.68)	116.29(113.64,118.94)	118.25(116.21,120.29)	< 0.001	< 0.001
**TPOAb(IU/mL)**	13.96(11.10,16.82)	18.89(9.01,28.77)	14.46(9.03,19.90)	8.48(4.34,12.62)	15.53(8.59,22.47)	0.12	0.268
**TgAb (IU/mL)**	7.68(4.07,11.29)	14.08(1.52,26.64)	5.94(2.26,9.62)	3.54(1.87, 5.21)	9.14(4.00,14.28)	0.06	0.742
**TSHL (mIU/L)**						0.07	0.133
> 1.07	73.89(66.69,81.08)	75.37(71.19,79.56)	77.96(73.68,82.24)	71.20(66.69,75.71)	71.28(67.73,74.84)		
< 1.07	26.11(22.80,29.43)	24.63(20.44,28.81)	22.04(17.76,26.32)	28.80(24.29,33.31)	28.72(25.16,32.27)		
**TSHH (mIU/L)**						0.08	0.446
< 2.33	74.90(67.99,81.81)	71.82(67.93,75.71)	71.90(66.91,76.88)	78.97(74.66,83.28)	76.62(72.32,80.91)		
> 2.33	25.10(21.69,28.51)	28.18(24.29,32.07)	28.10(23.12,33.09)	21.03(16.72,25.34)	23.38(19.09,27.68)		
**FT4L(pmol/L)**						0.02	0.191
> 9	86.33(78.78,93.88)	88.75(86.37,91.13)	81.96(77.55,86.37)	88.48(84.98,91.97)	85.89(81.67,90.12)		
< 9	13.67(10.67,16.68)	11.25(8.87,13.63)	18.04(13.63,22.45)	11.52(8.03,15.02)	14.11(9.88,18.33)		
**FT4H (pmol/L)**						0.15	0.013
< 11.5	75.09(66.57,83.62)	71.67(66.49,76.85)	75.07(70.66,79.48)	74.80(70.66,78.94)	78.81(73.90,83.71)		
> 11.5	24.91(21.84,27.97)	28.33(23.15,33.51)	24.93(20.52,29.34)	25.20(21.06,29.34)	21.19(16.29,26.10)		
**Thyroid**						0.04	
Subclinical hypothyroidism	1.58(0.97, 2.19)	1.16(0.34,1.99)	2.18(0.82,3.54)	1.36(0.43,2.28)	1.49(0.52,2.46)	0.50	
Subclinical hyperthyroidism	0.90(0.51, 1.29)	0.29(-0.03,0.62)	1.13(0.31,1.94)	0.71(0.25,1.17)	1.19(0.36,2.01)	0.38	
Overt hypothyroidism	2.32(1.52, 3.12)	1.16(0.47,1.85)	1.52(0.73,2.31)	2.42(0.70,4.14)	3.42(1.88,4.96)	0.02	
Overt hyperthyroidism	0.17(0, 0.34)	0.05(0,0.10)	0.57(0,1.34)	0.00(0.00,0.00)	0.04(0,0.08)	0.02	
AIT	18.23(15.52,20.94)	19.79(15.90,23.68)	18.21(14.42,22.00)	17.52(13.69,21.35)	18.66(15.00,22.33)	0.87	
**Thyroid Dysfunction**						0.08	
No	95.04(87.77,102.31)	97.34(96.07,98.60)	94.60(92.61,96.59)	95.52(93.73,97.30)	93.86(91.94,95.77)		
Yes	4.96(3.80, 6.12)	2.66(1.40,3.93)	5.40(3.41,7.39)	4.48(2.70,6.27)	6.14(4.23,8.06)		

All data was presented as mean (95% confidence interval). Participants had TSH quartiles of 1.57 (1.07–2.33) mIU/L and FT4 quartiles of 10.3 (9–11.5) pmol, TSHL refers to the low TSH quartile array, and TSHH refers to the high TSH quartile array; FT4L refers to the low FT4 quartile array, and FT4H refers to the high FT4 quartile array

## Discussion

In this cross-sectional study that included 5877 participants, we analyzed the PA and thyroid function of American adults from 2007 to 2012 and found that there was a significant sex difference in daily PA. The weekly PAM and PAT in men were higher than those in women. Our results from this large population study clearly show that thyroid function is closely related to PAM and PAT, and too high or too low MVPA levels have adverse effects mainly on male thyroid function. Although more women than men suffered from thyroid dysfunction, no significant differences were observed in women.

THs play an important role in the metabolic regulation and neurodevelopment of the human body [22]. We found that the levels of TSH and TT4 in men were lower than those in women, while the levels of FT3 and TT3 in men were higher than those in women, which was in line with the results of epidemiological investigations [23–25]. In addition, women were shown to have higher antibody levels than men and are more prone to thyroid dysfunction, which may be related to changes in estrogen in women [26]. It has been pointed out that estrogen receptor alpha activation contributes to the increased susceptibility of females to thyroid proliferative disorders and neoplastic transformation at least in part through the control of p27 levels [27]. Activated ERβ could directly bind to IL-17A and IL-21 gene promoters, and also indirectly promote IL-21 and RORγt gene transcription through interaction with NF-κB to stimulate the development of experimental autoimmune thyroiditis [28]. These studies all showed that estrogen may play an important role in thyroid disease.

While past studies have indicated that thyroid function in military personnel and athletes is strongly affected by prolonged PA [29], a recent study of a Dutch population found no relationship between thyroid function and PA levels [16]. Our study of American adults found that weekly PAM and PAT have certain effects on serum thyroid hormone levels (TSH, TT4, FT4, FT3, and TT3) within the normal range, and these effects occur mainly in relation to men. We found that men with higher PAM tended to have lower TSH and TT4 levels and higher FT3 levels, suggesting that increasing daily PA levels can affect corresponding changes in THs and thus may promote or suppress metabolic activities in other physiological systems. This result may also relate to increased T4 to T3 conversion by deiodinase in vivo. These findings are consistent with previous studies [30].

THs can also influence the levels of PA. Agitation is a characteristic of hyperthyroidism while hypothyroid patients tend to be less active [2]. TH receptors TRα and TRβ are both critical regulators of muscular genes and alterations in TRs are associated with muscle dysfunctions in mice and humans [31, 32]. In a study in 2023, Miro et al. have indicated that THs alter the proportion of saturated and unsaturated fatty acids (FAs) in Skeletal muscle (SKM) lipids membrane, and regulated the lipid content of muscle fibres, thereby affecting physical exercise performance [33]. TH plays a significant role in energy expenditure through both central and peripheral actions [34]. Muraca et al. indicated that resting energy expenditure (REE) decreases in obese women with hypothyroidism undergoing LT4 treatment [35]. PA can promote the body's energy expenditure and metabolic rate, but it remains unclear whether this process influences the body's energy metabolism balance through the modulation of THs. In addition, age may play an important role in the relationship between PA and THs. With age, PA is likely to decline and THs will change to some extent, it is worth exploring the effect of PA on the secretion and regulation of THs with further research.

We also found that men with relatively concentrated intervals of population density (PAM < 5000 MET*minutes/week, PAT < 1000 min/week) showed an L-shaped curve for TSH and an inverted U-shaped curve for FT4. In some studies, it has been noted that short periods of exercise can increase TSH, but we observed an L-shaped change in TSH, which may be related to the disturbance of homeostasis of thyroid hormone and prolactin in response to exercise stimulation [36]. Moderate TH fluctuations may be beneficial, depending on the delicate balance of the TH regulation process. Within the normal range, TSH stimulates the body's production of THs, which can regulate energy expenditure through the control of basal metabolic rate and thermogenesis [34]. This is an interesting dynamic regulatory process, and while TSH regulates thyroid hormones, plasma T3 and T4 may also negatively feedback regulate TSH [37]. TH stimulates basal metabolic rate(BMR) by increasing ATP production for metabolic processes and by generating and maintaining ion gradients, TH also could uncouple oxidative phosphorylation in the mitochondria to maintain BMR [34, 38]. One of the primary mechanisms through which thyroid hormones increase metabolic rate is by stimulating cellular respiration and energy production. T3 and T4 enhance the activity of mitochondria, the powerhouse of the cell, by increasing the expression of genes involved in oxidative phosphorylation and ATP synthesis and lead to an increase in BMR [34, 39]. The change in FT4 may be due to exercise-induced muscle adaptation and dynamic changes in type II and III deiodinases in vivo, and it is unclear whether it could be the result of increased hormone production or decreased uptake by target tissues [40–42]. Notably, the changes in FT3 and TT3 in men are linearly positively correlated with PAM and PAT, while TT4 is linearly negatively correlated. Exercise increases SKM type 2 deiodinase expression through a beta-adrenergic receptor-dependent mechanism [42]. The type 2 deiodinase (D2) can catalyze the conversion of the prohormone thyroxine (T4) into T3, and the accelerated conversion of T4 to T3 in myocytes promotes peroxisome proliferator-activated receptor-γ coactivator-1α (PGC-1a) expression, which can mediate mitochondrial biogenesis and oxidative capacity in SKM [43]. This might explain the relation between FT3, Total T4 and Total T3 appears to bi linear. FT3 is the most sensitive indicator in the diagnosis of hyperthyroidism, and its results indicate that PA may be closely related to the risk of hyperthyroidism [44]. Whether the changes in TT3 and TT4 involve some mechanism of hormonal pathway changes requires further experimental research. On the other hand, steroid hormones may have the potential influence on the results. Steroid hormones, such as cortisol and sex hormones, play significant roles in regulating metabolism, immune function, and various physiological processes [45]. Fluctuations in cortisol levels, for example, due to stress or exercise, may impact thyroid function and metabolic rate [46]. There may exist a complex interplay between sex hormones and THs about the metabolic homeostasis.

Our results show that thyroid function is strongly affected by higher PAM and PAT, and there is a non-linear relationship between PA and thyroid disease. High-intensity PA may impair thyroid function by dysregulating the thyroid gland, which plays a key role during and after PA [47]. Fiore noted that increasing daily walking time may reduce the risk of thyroid cancer [48, 49], and Wu found that exercise is closely related to thyroid homeostasis in patients with SCH [50]. Our logistic regression analysis revealed that PAM and PAT were associated with hyperthyroidism, overt hypothyroidism, and AIT in men, while studies have shown that exercise has no relationship with hyperthyroidism. Huang concluded that regular PA may be a protective factor against hypothyroidism [51], while we found an interesting result, that is, the correlation between PA and overt hypothyroidism in men showed a nonlinear J-shaped curve when PA levels were within the categories of PAM<5000 MET*minutes/week and PAT<1000 minutes/week. This result may be attributed to a delayed response of the thyroid gland to hormonal alterations in the body, followed by adaptive changes. This finding is conducive to providing an appropriate reference range of PA for male patients with hypothyroidism.

AIT is a common autoimmune disease, and research shows that thyroid autoimmunity itself has no relationship with the level of PA, but Hashimoto thyroiditis may lead to muscle weakness due to hypothyroid dysfunction [52, 53]. We observed a U-shaped curve of the correlation between AIT and PA in men at PAT < 1000 min/week and PAM < 5000 MET*minutes/week, and the U-shaped RCS curve for AIT may indicate that there is an appropriate range of PA for AIT patients. This also means that we need to determine the optimal range of PA levels for AIT patients based on the risk of AIT. Given that PA may be limited due to hypothyroid dysfunction in patients with AIT after the disease [53, 54], we suggest that patients with AIT have a greater need to increase their PA levels. In addition, previous findings found that cannabis consumption is associated with a high level of PA [55]. Our research also confirms that current smokers account for a large proportion of the high-PA group, which may be related to the neurological effects of nicotine in the body and the stress of life and work.

Our analysis shows that PA has an impact on the changes in the thyroid biochemical indicators TSH, FT4, TT4, FT3, and TT3 when they are within the normal range, which can provide a reference for the influence of PA on the changes in clinical thyroid function laboratory test indicators. We provided data for both PAM and PAT, which can make these results more accurate and comprehensive. Our study may potentially be useful for developing appropriate activity guidelines for people with thyroid dysfunction. There are also some limitations in our study. One important limitation is the potential for information bias in NHANES questionnaires, which may lead to underreporting or overreporting of PA and sedentary behavior levels by participants. This may affect the accuracy of the data collected. Notably, this study is cross-sectional in nature, which limits our ability to explore the long-term effects of PA on thyroid diseases. While our study identified associations between PA and thyroid function, we cannot distinct the direction of the association between PA and THs. Reverse causality may also be an explanation for the detected associations here, since thyroid hormone levels may also affect the PA of an individual. To fully understand the specific mechanisms underlying the observed associations, further experimental studies and longitudinal cohort studies are needed. PA is not normally distributed, and the use of quartiles allows for a more comprehensive representation of the diverse PA ranges observed. The huge range within the higher quartiles potentially may dilute or mask the potential effects within the lower quartiles is well-taken. In addition, the low number of men with hyperthyroidism in the study may have led to false-positive results when assessing the relationship between hyperthyroidism and PA in men.

In conclusion, PA has a certain effect on thyroid-related biochemical parameters and thyroid diseases in US adults. Quantification and qualification based on individual circumstances should be accounted for when engaging in PA to avoid excessive exercise. Our study highlights the importance of maintaining a balanced level of PA to promote optimal thyroid function and prevent the development of thyroid diseases. Future research should focus on elucidating the underlying mechanisms and developing personalized exercise recommendations for individuals with or at risk for thyroid diseases.

### Supplementary Information

Supplementary Materials.

## Data Availability

Data are available from the NHANES (NHANES—National Health and Nutrition Examination Survey Homepage (cdc.gov)) in 2007–2012.

## Abbreviations

AIT: Autoimmune thyroiditis
BMR: Basal metabolic rate
D2: Type 2 deiodinase
FAs: Fatty acids
MVPA: Moderate-to-vigorous-intensity physical activity
NHANES: The National Health and Nutrition Examination Survey
PA: Physical activity
PAM: Weekly physical activity, expressed in metabolic equivalents of task
PAT: Physical activity time
PGC‐1a: Peroxisome proliferator‐activated receptor‐γ coactivator‐1α
SKM: Skeletal muscle
THs: Thyroid hormones
T3: 3,5,3′‐Triiodothyronine
T4: Thyroxine

## References

[1] Cheng S-Y, Leonard JL, Davis PJ (2010). Molecular aspects of thyroid hormone actions. Endocr Rev.

[2] Taylor PN, Albrecht D, Scholz A, Gutierrez-Buey G, Lazarus JH, Dayan CM (2018). Global epidemiology of hyperthyroidism and hypothyroidism. Nat Rev Endocrinol.

[3] Teng W, Shan Z, Teng X, Guan H, Li Y, Teng D (2006). Effect of iodine intake on thyroid diseases in China. N Engl J Med.

[4] Zimmermann MB, Boelaert K (2015). Iodine deficiency and thyroid disorders. Lancet Diabetes Endocrinol.

[5] Biondi B, Cappola AR, Cooper DS (2019). Subclinical hypothyroidism: a review. JAMA.

[6] Hollowell JG, Staehling NW, Flanders WD, Hannon WH, Gunter EW, Spencer CA (2002). Serum Tsh, T(4), and Thyroid Antibodies in the United States Population (1988 to 1994): National Health and Nutrition Examination Survey (Nhanes Iii). J Clin Endocrinol Metab.

[7] Caspersen CJ, Powell KE, Christenson GM (1985). Physical activity, exercise, and physical fitness: definitions and distinctions for health-related research. Public Health Rep.

[8] Piercy KL, Troiano RP, Ballard RM, Carlson SA, Fulton JE, Galuska DA (2018). The physical activity guidelines for Americans. JAMA.

[9] Tucker LA (2017). Physical activity and telomere length in U.S. men and women: an nhanes investigation. Prev Med.

[10] Carlson SA, Adams EK, Yang Z, Fulton JE (2018). Percentage of deaths associated with inadequate physical activity in the United States. Prev Chronic Dis.

[11] Saint-Maurice PF, Graubard BI, Troiano RP, Berrigan D, Galuska DA, Fulton JE (2022). Estimated number of deaths prevented through increased physical activity among us adults. JAMA Intern Med.

[12] Xu F, Earp JE, Adami A, Weidauer L, Greene GW. The Relationship of Physical Activity and Dietary Quality and Diabetes Prevalence in Us Adults: Findings from Nhanes 2011–2018. Nutrients. 2022;14(16). 10.3390/nu14163324. 10.3390/nu14163324 PMC9414710 36014830

[13] Aakre KM, Omland T (2019). Physical activity, exercise and cardiac troponins: clinical implications. Prog Cardiovasc Dis.

[14] Aengevaeren VL, Baggish AL, Chung EH, George K, Kleiven Ø, Mingels AMA (2021). Exercise-induced cardiac troponin elevations: from underlying mechanisms to clinical relevance. Circulation.

[15] Byeon H. Relationship between physical activity level and depression of elderly people living alone. Int J Environ Res Public Health. 2019;16(20). 10.3390/ijerph16204051. 10.3390/ijerph16204051 PMC6843978 31652619

[16] Roa Dueñas OH, Koolhaas C, Voortman T, Franco OH, Ikram MA, Peeters RP (2021). Thyroid function and physical activity: a population-based cohort study. Thyroid.

[17] Cooper DS, Biondi B (2012). Subclinical thyroid disease. Lancet.

[18] Biondi B, Cooper DS (2018). Subclinical hyperthyroidism. N Engl J Med.

[19] Vanderpump MPJ (2011). The epidemiology of thyroid disease. Br Med Bull.

[20] Health UDo, Services H. Physical Activity Guidelines for Americans: Be Active, Healthy, and Happy!. http://www.health.gov/paguidelines/guidelines/default aspx. 2008.

[21] Singh R, Pattisapu A, Emery MS (2020). Us physical activity guidelines: current state, impact and future directions. Trends Cardiovasc Med.

[22] Brent GA (2012). Mechanisms of thyroid hormone action. J Clin Invest.

[23] Santos-Palacios S, Brugos-Larumbe A, Guillén-Grima F, Galofré JC (2013). A cross-sectional study of the association between circulating tsh level and lipid profile in a large spanish population. Clin Endocrinol (Oxf).

[24] Wang D, Wan S, Liu P, Meng F, Zhang X, Ren B (2021). Relationship between excess iodine, thyroid function, blood pressure, and blood glucose level in adults, pregnant women, and lactating women: a cross-sectional study. Ecotoxicol Environ Saf.

[25] Lee JJ, Pedley A, Marqusee E, Sutherland P, Hoffmann U, Massaro JM (2016). Thyroid function and cardiovascular disease risk factors in euthyroid adults: a cross-sectional and longitudinal study. Clin Endocrinol (Oxf).

[26] Merrheim J, Villegas J, Van Wassenhove J, Khansa R, Berrih-Aknin S, le Panse R (2020). Estrogen, estrogen-like molecules and autoimmune diseases. Autoimmun Rev.

[27] Antico-Arciuch VG, Dima M, Liao XH, Refetoff S, Di Cristofano A (2010). Cross-talk between Pi3k and estrogen in the mouse thyroid predisposes to the development of follicular carcinomas with a higher incidence in females. Oncogene.

[28] Qin J, Li L, Jin Q, Guo D, Liu M, Fan C (2018). Estrogen receptor Β activation stimulates the development of experimental autoimmune thyroiditis through up-regulation of Th17-Type responses. Clin Immunol.

[29] Opstad PK, Falch D, Oktedalen O, Fonnum F, Wergeland R (1984). The thyroid function in young men during prolonged exercise and the effect of energy and sleep deprivation. Clin Endocrinol (Oxf).

[30] Klasson CL, Sadhir S, Pontzer H (2022). Daily physical activity is negatively associated with thyroid hormone levels, inflammation, and immune system markers among men and women in the Nhanes dataset. PLoS ONE.

[31] Nappi A, Murolo M, Cicatiello AG, Sagliocchi S, Di Cicco E, Raia M, et al. Thyroid hormone receptor isoforms alpha and beta play convergent roles in muscle physiology and metabolic regulation. Metabolites. 2022;12(5). 10.3390/metabo12050405. 10.3390/metabo12050405 PMC9145723 35629909

[32] Milanesi A, Lee J-W, Yang A, Liu Y-Y, Sedrakyan S, Cheng S-Y (2017). Thyroid hormone receptor alpha is essential to maintain the satellite cell niche during skeletal muscle injury and sarcopenia of aging. Thyroid.

[33] Miro C, Nappi A, Sagliocchi S, Di Cicco E, Murolo M, Torabinejad S, et al. Thyroid hormone regulates the lipid content of muscle fibers, thus affecting physical exercise performance. Int J Mol Sci. 2023;24(15). 10.3390/ijms241512074. 10.3390/ijms241512074 PMC10418733 37569453

[34] Mullur R, Liu Y-Y, Brent GA (2014). Thyroid hormone regulation of metabolism. Physiol Rev.

[35] Muraca E, Ciardullo S, Oltolini A, Zerbini F, Bianconi E, Perra S, et al. Resting energy expenditure in obese women with primary hypothyroidism and appropriate levothyroxine replacement therapy. J Clin Endocrinol Metabol. 2020;105(4). 10.1210/clinem/dgaa097. 10.1210/clinem/dgaa097 32119074

[36] Di Blasio A, Di Dalmazi G, Izzicupo P, Bucci I, Giuliani C, Di Baldassarre A, et al. Serum Tsh and daily physical activity in a cohort of nonagenarians: results from the mugello study. J Funct Morphol Kinesiol. 2022;7(3). 10.3390/jfmk7030056. 10.3390/jfmk7030056 PMC9396985 35997372

[37] Fonseca TL, Correa-Medina M, Campos MP, Wittmann G, Werneck-de-Castro JP, Arrojo e Drigo R (2013). Coordination of hypothalamic and pituitary T3 production regulates Tsh expression. J Clin Invest.

[38] Bianco AC, Dumitrescu A, Gereben B, Ribeiro MO, Fonseca TL, Fernandes GW (2019). Paradigms of dynamic control of thyroid hormone signaling. Endocr Rev.

[39] Cioffi F, Giacco A, Goglia F, Silvestri E. Bioenergetic aspects of mitochondrial actions of thyroid hormones. Cells. 2022;11(6). 10.3390/cells11060997. 10.3390/cells11060997 PMC8947633 35326451

[40] Philippou A, Maridaki M, Tenta R, Koutsilieris M (2017). Hormonal responses following eccentric exercise in humans. Hormones (Athens).

[41] Dentice M, Marsili A, Ambrosio R, Guardiola O, Sibilio A, Paik J-H (2010). The Foxo3/Type 2 deiodinase pathway is required for normal mouse myogenesis and muscle regeneration. J Clin Invest.

[42] Salvatore D, Simonides WS, Dentice M, Zavacki AM, Larsen PR (2014). Thyroid hormones and skeletal muscle-new insights and potential implications. Nat Rev Endocrinol.

[43] Bocco BMLC, Louzada RAN, Silvestre DHS, Santos MCS, Anne-Palmer E, Rangel IF (2016). Thyroid Hormone Activation by Type 2 Deiodinase Mediates Exercise-Induced Peroxisome Proliferator-Activated Receptor-Γ Coactivator-1α Expression in Skeletal Muscle. J Physiol.

[44] Hackney AC, Saeidi A (2019). The thyroid axis, prolactin, and exercise in humans. Curr Opin Endocr Metab Res.

[45] Falkenstein E, Tillmann HC, Christ M, Feuring M, Wehling M (2000). Multiple actions of steroid hormones–a focus on rapid. Nongenomic Effects Pharmacol Rev.

[46] Katz FH, Kappas A (1967). The effects of estradiol and estriol on plasma levels of cortisol and thyroid hormone-binding globulins and on aldosterone and cortisol secretion rates in man. J Clin Invest.

[47] Phillips KJ. Beige fat, adaptive thermogenesis, and its regulation by exercise and thyroid hormone. Biology (Basel). 2019;8(3). 10.3390/biology8030057. 10.3390/biology8030057 PMC6783838 31370146

[48] Fiore M, Cristaldi A, Okatyeva V, Lo Bianco S, Oliveri Conti G, Zuccarello P, et al. Physical activity and thyroid cancer risk: a case-control study in Catania (South Italy). Int J Environ Res Public Health. 2019;16(8). 10.3390/ijerph16081428. 10.3390/ijerph16081428 PMC6517930 31013573

[49] Bui AQ, Gunathilake M, Lee J, Lee EK, Kim J (2022). Relationship between physical activity levels and thyroid cancer risk: a prospective cohort study in Korea. Thyroid.

[50] Wu K, Zhou Y, Ke S, Huang J, Gao X, Li B (2021). Lifestyle is associated with thyroid function in subclinical hypothyroidism: a cross-sectional study. BMC Endocr Disord.

[51] Huang Y, Cai L, Zheng Y, Pan J, Li L, Zong L (2019). Association between lifestyle and thyroid dysfunction: a cross-sectional epidemiologic study in the she ethnic minority group of Fujian Province in China. BMC Endocr Disord.

[52] Siciliano G, Monzani F, Manca ML, Tessa A, Caraccio N, Tozzi G (2002). Human mitochondrial transcription factor a reduction and mitochondrial dysfunction in Hashimoto's hypothyroid myopathy. Mol Med.

[53] Jordan B, Uer O, Buchholz T, Spens A, Zierz S (2021). Physical fatigability and muscle pain in patients with Hashimoto thyroiditis. J Neurol.

[54] Lankhaar JAC, Kemler E, Hofstetter H, Collard DCM, Zelissen PMJ, Stubbe JH (2021). Physical activity, sports participation and exercise-related constraints in adult women with primary hypothyroidism treated with thyroid hormone replacement therapy. J Sports Sci.

[55] Smith L, Sherratt F, Barnett Y, Cao C, Tully MA, Koyanagi A (2021). Physical activity, sedentary behaviour and cannabis use in 15,822 Us adults: cross-sectional analyses from Nhanes. Public Health.

