# Supplementary material for: Association between physical activity and thyroid function in American adults: a survey from the NHANES database
Source: BMC Public Health. 2024 May 10;24:1277. doi: 10.1186/s12889-024-18768-4 (PMC11084014; doi:10.1186/s12889-024-18768-4)
Supplement: Supplementary file 1 — Supplementary Materials. [file 12889_2024_18768_MOESM1_ESM.docx]

**Supplements:**

**Table S1:** Supplementary table of multivariate correction information, NHANES 2007-2012.

| **PAT** |  |  | **SHYPER** | | **HYPER** | | **SHYPO** | | **HYPO** | | **AIT** | | |  |
| --- | --- | --- | --- | --- | --- | --- | --- | --- | --- | --- | --- | --- | --- | --- |
|  |  |  | **P** | **OR (95%CI)** | **P** | **OR (95%CI)** | **P** | **OR (95%CI)** | **P** | **OR (95%CI)** | | **P** | **OR (95%CI)** | |
|  |  | Q1 | ref | ref | ref | ref | ref | ref | ref | ref | | ref | ref | |
|  | Model1 | Q2 | 0.23 | 0.58(0.24,1.43) | 0.24 | 0.64(0.29,1.38) | 0.52 | 0.74(0.28,1.91) | 0.07 | 2.11(0.94,4.72) | | 0.88 | 1.03(0.72,1.46) | |
|  |  | Q3 | 0.39 | 0.64(0.23,1.80) | 0.38 | 0.67(0.27,1.67) | 0.25 | 1.68(0.68,4.13) | 0.4 | 0.62(0.20,1.92) | | 0.89 | 1.03(0.70,1.50) | |
|  |  | Q4 | 0.1 | 0.45(0.18,1.17) | 0.06 | 0.41(0.16,1.02) | 0.96 | 1.02(0.39,2.70) | 0.6 | 1.31(0.47,3.59) | | 0.89 | 0.97(0.65,1.46) | |
|  |  | Q1 | ref | ref | ref | ref | ref | ref | ref | ref | | ref | ref | |
|  | Model2 | Q2 | 0.2 | 0.62(0.29,1.30) | 0.73 | 1.06(0.74,1.52) | 0.72 | 1.17(0.49,2.79) | 0.17 | 1.84(0.76,4.45) | | 0.73 | 1.06(0.74,1.52) | |
| **Female** |  | Q3 | 0.94 | 0.96(0.34,2.70) | 0.59 | 1.11(0.76,1.62) | 0.79 | 1.14(0.44,2.98) | 0.09 | 0.37(0.12,1.18) | | 0.59 | 1.11(0.76,1.62) | |
|  |  | Q4 | 0.12 | 0.44(0.16,1.25) | 0.6 | 1.12(0.73,1.70) | 0.72 | 0.83(0.30,2.29) | 0.38 | 1.62(0.54,4.91) | | 0.6 | 1.12(0.73,1.70) | |
|  |  | Q1 | ref | ref | ref | ref | ref | ref | ref | ref | | ref | ref | |
|  | Model3 | Q2 | 0.2 | 0.61(0.28,1.32) | 0.84 | 0.85(0.16, 4.49) | 0.72 | 1.17(0.48,2.88) | 0.2 | 1.78(0.73,4.30) | | 0.89 | 1.03(0.71,1.48) | |
|  |  | Q3 | 0.91 | 0.94(0.32,2.73) | 0.79 | 0.81(0.17, 3.94) | 0.79 | 1.14(0.42,3.09) | 0.07 | 0.36(0.12,1.11) | | 0.77 | 1.06(0.71,1.57) | |
|  |  | Q4 | 0.11 | 0.43(0.15,1.23) | 0.09 | 0.13(0.01, 1.35) | 0.65 | 0.80(0.30,2.17) | 0.34 | 1.70(0.56,5.20) | | 0.64 | 1.11(0.71,1.72) | |
| **PAM** |  |  |  |  |  |  |  |  |  |  | |  |  | |
|  |  | Q1 | ref | ref | ref | ref | ref | ref | ref | ref | | ref | ref | |
|  | Model1 | Q2 | 0.4 | 0.69(0.29,1.65) | 0.49 | 0.77(0.36,1.63) | 0.74 | 1.17(0.45,3.03) | 0.16 | 1.85(0.78,4.41) | | 0.97 | 0.99(0.69,1.43) | |
|  |  | Q3 | 0.46 | 0.69(0.25,1.88) | 0.37 | 0.66(0.26,1.66) | 0.48 | 1.36(0.57,3.27) | 0.66 | 0.81(0.32,2.07) | | 0.76 | 0.95(0.65,1.36) | |
|  |  | Q4 | 0.08 | 0.39(0.14,1.12) | 0.06 | 0.39(0.15,1.03) | 0.57 | 0.76(0.30,1.96) | 0.64 | 1.26(0.47,3.33) | | 0.78 | 0.95(0.65,1.38) | |
|  |  | Q1 | ref | ref | ref | ref | ref | ref | ref | ref | | ref | ref | |
|  | Model2 | Q2 | 0.4 | 0.68(0.28,1.69) | 0.69 | 1.39(0.27,7.30) | 0.73 | 1.17(0.46,2.97) | 0.15 | 1.95(0.78,4.88) | | 0.87 | 1.03(0.70,1.51) | |
| **Female** |  | Q3 | 0.54 | 0.72(0.24,2.11) | 0.43 | 0.49(0.08,2.95) | 0.42 | 1.39(0.61,3.16) | 0.77 | 0.87(0.34,2.23) | | 0.87 | 1.03(0.71,1.49) | |
|  |  | Q4 | 0.12 | 0.45(0.16,1.25) | 0.47 | 0.51(0.08,3.30) | 0.64 | 0.81(0.32,2.04) | 0.6 | 1.32(0.46,3.77) | | 0.58 | 1.11(0.75,1.64) | |
|  |  | Q1 | ref | ref | ref | ref | ref | ref | ref | ref | | ref | ref | |
|  | Model3 | Q2 | 0.45 | 0.70(0.28,1.78) | 0.61 | 1.53(0.28, 8.32) | 0.75 | 1.16(0.45,2.97) | 0.15 | 1.94(0.78,4.87) | | 1 | 1.00(0.68,1.48) | |
|  |  | Q3 | 0.52 | 0.71(0.24,2.11) | 0.36 | 0.44(0.08, 2.62) | 0.49 | 1.32(0.58,2.99) | 0.73 | 0.85(0.34,2.15) | | 0.94 | 0.99(0.67,1.45) | |
|  |  | Q4 | 0.11 | 0.45(0.16,1.21) | 0.39 | 0.45(0.07, 2.84) | 0.52 | 0.75(0.30,1.87) | 0.55 | 1.37(0.48,3.91) | | 0.63 | 1.10(0.74,1.64) | |

ORs are estimated with logistic models. P value less than 0.05 means statistically significant.

Table S2: Relationship between thyroid function and PAM in American adults aged 18-85 years, NHANES 2007-2012.

| **Variable** | **Total** | **<840MET-minutes/week** | **840-2400MET-minutes/week** | **2400-5960MET-minutes/week** | **>5960MET-minutes/week** | **P value** | **P for**  **trend** |
| --- | --- | --- | --- | --- | --- | --- | --- |
| **Age** **(yr)** **^a^** | 44.07(0.43) | 47.94(0.47) | 45.84(0.45) | 42.80(0.78) | 39.98(0.60) | <0.0001 |  |
| **BMI** | 28.02(0.13) | 28.95(0.19) | 27.82(0.20) | 27.53(0.23) | 27.88(0.17) | <0.0001 |  |
| **TSH** **(mIU/L)** **^a^** | 2.03(0.05) | 2.22(0.14) | 2.15(0.15) | 1.98(0.08) | 1.79(0.05) | 0.002 | 0.007 |
| **FT4(pmol/L)** **^a^** | 10.23(0.07) | 10.35(0.07) | 10.31(0.09) | 10.24(0.09) | 10.02(0.08) | <0.0001 | <0.001 |
| **FT3(pg/mL)** **^a^** | 3.20(0.01) | 3.14(0.01) | 3.15(0.01) | 3.23(0.02) | 3.28(0.01) | <0.0001 | <0.0001 |
| **TT4(ug/dL)** **^a^** | 7.78(0.04) | 7.99(0.06) | 7.85(0.06) | 7.69(0.05) | 7.60(0.06) | <0.0001 | <0.0001 |
| **TT3(ng/dl)** **^a^** | 114.53(0.66) | 112.75(0.95) | 111.99(0.80) | 115.58(1.03) | 117.72(0.92) | <0.0001 | <0.0001 |
| **TPOAb** **(IU/mL)** **^a^** | 21.73(1.11) | 23.76(3.01) | 26.22(2.80) | 22.23(3.42) | 14.66(2.35) | 0.02 | 0.008 |
| **TgAb** **(IU/mL)** **^a^** | 9.95(1.38) | 13.40(3.29) | 9.03(2.33) | 6.24(1.88) | 11.52(2.33) | 0.17 | 0.839 |
| **TSHL** **(mIU/L)** **^b^** |  |  |  |  |  | 0.09 | 0.021 |
| > 1.07 | 75.28(68.64,81.93) | 78.04(74.87,81.21) | 76.12(73.56,78.67) | 73.79(70.99,76.59) | 73.38(70.80,75.97) |  |  |
| < 1.07 | 24.72(22.65,26.78) | 21.96(18.79,25.13) | 23.88(21.33,26.44) | 26.21(23.41,29.01) | 26.62(24.03,29.20) |  |  |
| **TSHH** **(mIU/L)** **^b^** |  |  |  |  |  | 0.09 | 0.022 |
| <2.33 | 75.15(69.57,80.73) | 72.03(68.88,75.19) | 75.48(72.30,78.67) | 74.99(71.83,78.15) | 77.88(74.74,81.02) |  |  |
| >2.33 | 24.85(21.73,27.96) | 27.97(24.81,31.12) | 24.52(21.33,27.70) | 25.01(21.85,28.17) | 22.12(18.98,25.26) |  |  |
| **FT4L(pmol/L)** **^b^** |  |  |  |  |  | 0.29 | 0.233 |
| >9 | 87.15(80.60,93.71) | 88.42(86.38,90.47) | 86.07(83.51,88.63) | 87.92(85.27,90.57) | 86.30(83.21,89.39) |  |  |
| <9 | 12.85(10.32,15.38) | 11.58(9.53,13.62) | 13.93(11.37,16.49) | 12.08(9.43,14.73) | 13.70(10.61,16.79) |  |  |
| **FT4H** **(pmol/L)** **^b^** |  |  |  |  |  | 0.08 | 0.054 |
| < 11.5 | 74.32(66.56,82.08) | 72.97(69.70,76.24) | 73.20(69.69,76.71) | 73.32(69.02,77.62) | 77.67(73.89,81.46) |  |  |
| > 11.5 | 25.68(23.08,28.28) | 27.03(23.76,30.30) | 26.80(23.29,30.31) | 26.68(22.38,30.98) | 22.33(18.54,26.11) |  |  |
| **Sex ^b^** |  |  |  |  |  | <0.0001 |  |
| Male | 51.17(47.13,55.22) | 36.40(34.27,38.53) | 46.28(43.57,48.99) | 54.17(51.15,57.19) | 66.88(63.69,70.08) |  |  |
| Female | 48.83(44.23,53.43) | 63.60(61.47,65.73) | 53.72(51.01,56.43) | 45.83(42.81,48.85) | 33.12(29.92,36.31) |  |  |
| **Race ^b^** |  |  |  |  |  | 0.004 |  |
| Mexican American | 7.69(6.31,9.08) | 7.18(5.35,9.02) | 6.86(4.80,8.93) | 7.19(5.49,8.89) | 9.54(7.67,11.4) |  |  |
| Non-Hispanic Black | 9.55(7.65,11.4) | 10.75(8.42,13.8) | 8.07(6.05,10.10) | 9.16(6.91,11.41) | 10.38(7.40,13.36) |  |  |
| Non-Hispanic White | 71.51(62.32,80.70) | 70.85(67.08,74.61) | 72.77(68.24,77.30) | 72.64(68.64,76.64) | 69.65(63.46,75.84) |  |  |
| Other Hispanic | 4.79(3.53, 6.05) | 4.49(3.22,5.77) | 4.16(2.82,5.50) | 4.85(3.66,6.04) | 5.66(3.52,7.81) |  |  |
| Other Race - Including  Multi-Racial | 6.45(5.14, 7.76) | 6.73(4.55, 8.91) | 8.13(5.96,10.31) | 6.16(4.48, 7.85) | 4.76(2.65, 6.87) |  |  |
| **Education ^b^** |  |  |  |  |  | <0.0001 |  |
| < University | 36.95(32.68,41.23) | 38.64(34.24,43.04) | 32.68(28.18,37.19) | 33.90(30.52,37.28) | 49.31(44.67,53.95) |  |  |
| >= University | 59.00(53.06,64.93) | 61.36(56.96,65.76) | 67.32(62.81,71.82) | 66.10(62.72,69.48) | 50.69(46.05,55.33) |  |  |
| **Income ^b^** |  |  |  |  |  | 0.14 |  |
| < $20000 | 16.53(14.37,18.68) | 17.93(15.05,20.81) | 15.42(12.65,18.18) | 16.07(13.15,19.00) | 18.89(15.32,22.46) |  |  |
| >$20000 | 80.42(72.65,88.19) | 82.07(79.19,84.95) | 84.58(81.82,87.35) | 83.93(81.00,86.85) | 81.11(77.54,84.68) |  |  |
| **Smoke ^b^** |  |  |  |  |  | <0.0001 |  |
| Never smoker | 52.30(47.50,57.10) | 54.72(51.96,57.48) | 60.53(57.15,63.91) | 53.48(49.17,57.80) | 48.92(44.82,53.02) |  |  |
| Former smoker | 23.53(21.28,25.78) | 26.41(23.49,29.32) | 24.97(22.24,27.70) | 26.08(22.81,29.36) | 20.61(17.90,23.33) |  |  |
| Current smoker | 20.16(17.86,22.46) | 18.88(16.61,21.14) | 14.50(12.19,16.81) | 20.43(17.54,23.33) | 30.47(27.06,33.88) |  |  |
| **Thyroid ^b^** |  |  |  |  |  | 0.2 |  |
| Normal | 93.81(86.37,101.26) | 93.87(92.19,95.54) | 93.31(91.75,94.86) | 93.70(92.04,95.35) | 94.41(92.76,96.07) |  |  |
| Subclinical hypothyroidism | 2.05(1.50, 2.60) | 1.84(0.98,2.71) | 2.26(1.39,3.14) | 2.83(1.61,4.06) | 1.22(0.44,1.99) |  |  |
| Subclinical hyperthyroidism | 1.52(1.11, 1.92) | 1.91(1.00,2.81) | 1.41(0.62,2.20) | 1.44(0.69,2.18) | 1.34(0.62,2.06) |  |  |
| Overt hypothyroidism | 2.37(1.82, 2.92) | 2.06(1.11,3.00) | 2.53(1.72,3.35) | 1.90(0.98,2.81) | 2.98(1.90,4.06) |  |  |
| Overt hyperthyroidism | 0.25(0.10, 0.40) | 0.32(0.04,0.60) | 0.48(0.03,0.93) | 0.14(-0.02,0.29) | 0.05(-0.02,0.11) |  |  |
| AIT | 13.78(12.05,15.51) | 15.41(12.74,18.08) | 14.54(12.11,16.96) | 12.97(10.40,15.53) | 12.97(10.69,15.25) | 0.47 |  |

a: presented as mean ± SEM; b: presented as 95% confidence interval. P value less than 0.05 means statistically significant.

**Table S3:** Relationship between thyroid function and PAT in American adults aged 18-85 years, NHANES 2007-2012.

| **Variable** | **Total** | **<180**  **minutes/week** | **180-480 minutes/week** | **480-1200 minutes/week** | **>1200**  **minutes/week** | **P**  **value** | **P for trend** |
| --- | --- | --- | --- | --- | --- | --- | --- |
| **Age** **(yr)** **^a^** | 44.07(0.43) | 47.02(0.55) | 45.47(0.44) | 43.57(0.76) | 40.57(0.62) | <0.0001 |  |
| **BMI** **^a^** | 28.02(0.13) | 28.86(0.20) | 27.77(0.17) | 27.56(0.25) | 28.02(0.17) | < 0.0001 |  |
| **TSH** **(mIU/L)** **^a^** | 2.03(0.05) | 2.21(0.15) | 2.16(0.15) | 1.95(0.08) | 1.83(0.05) | 0.02 | 0.026 |
| **FT4(pmol/L)** **^a^** | 10.23(0.07) | 10.34(0.08) | 10.33(0.08) | 10.25(0.09) | 10.01(0.08) | < 0.001 | 0.002 |
| **FT3(pg/mL)** **^a^** | 3.20(0.01) | 3.16(0.01) | 3.14(0.01) | 3.23(0.02) | 3.27(0.01) | < 0.0001 | <0.0001 |
| **TT4(ug/dL)** **^a^** | 7.78(0.04) | 7.98(0.07) | 7.83(0.04) | 7.72(0.06) | 7.60(0.05) | < 0.0001 | <0.0001 |
| **TT3(ng/dl)** **^a^** | 114.53(0.66) | 112.63(0.99) | 112.62(0.80) | 115.34(1.07) | 117.42(0.91) | < 0.0001 | <0.0001 |
| **TPOAb** **(IU/mL)** **^a^** | 20.90(1.10) | 23.17(3.10) | 25.31(2.73) | 21.66(3.63) | 13.58(1.84) | 0.002 | 0.032 |
| **TgAb(IU/mL) ^a^** | 8.95(1.07) | 11.31(2.25) | 6.48(1.49) | 6.33(2.02) | 11.83(2.55) | 0.12 | 0.438 |
| **TSHL** **(mIU/L) ^b^** |  |  |  |  |  | 0.09 | 0.034 |
| > 1.07 | 75.28(68.64,81.93) | 78.04(74.87,81.21) | 76.12(73.56,78.67) | 73.79(70.99,76.59) | 73.38(70.80,75.97) |  |  |
| < 1.07 | 24.72(22.65,26.78) | 21.96(18.79,25.13) | 23.88(21.33,26.44) | 26.21(23.41,29.01) | 26.62(24.03,29.20) |  |  |
| **TSHH** **(mIU/L)** **^b^** |  |  |  |  |  | 0.09 | 0.076 |
| <2.33 | 75.15(69.57,80.73) | 72.03(68.88,75.19) | 75.48(72.30,78.67) | 74.99(71.83,78.15) | 77.88(74.74,81.02) |  |  |
| >2.33 | 24.85(21.73,27.96) | 27.97(24.81,31.12) | 24.52(21.33,27.70) | 25.01(21.85,28.17) | 22.12(18.98,25.26) |  |  |
| **FT4L(pmol/L)** **^b^** |  |  |  |  |  | 0.29 | 0.382 |
| >9 | 87.15(80.60,93.71) | 88.42(86.38,90.47) | 86.07(83.51,88.63) | 87.92(85.27,90.57) | 86.30(83.21,89.39) |  |  |
| <9 | 12.85(10.32,15.38) | 11.58(9.53,13.62) | 13.93(11.37,16.49) | 12.08(9.43,14.73) | 13.70(10.61,16.79) |  |  |
| **FT4H** **(pmol/L)** **^b^** |  |  |  |  |  | 0.08 | 0.022 |
| < 11.5 | 74.32(66.56,82.08) | 72.97(69.70,76.24) | 73.20(69.69,76.71) | 73.32(69.02,77.62) | 77.67(73.89,81.46) |  |  |
| > 11.5 | 25.68(23.08,28.28) | 27.03(23.76,30.30) | 26.80(23.29,30.31) | 26.68(22.38,30.98) | 22.33(18.54,26.11) |  |  |
| **Sex ^b^** |  |  |  |  |  | < 0.0001 |  |
| Male | 51.17(47.13,55.22) | 38.02(36.13,39.91) | 46.03(43.52,48.54) | 54.74(51.50,57.98) | 64.44(61.34,67.53) |  |  |
| Female | 48.83(44.23,53.43) | 61.98(60.09,63.87) | 53.97(51.46,56.48) | 45.26(42.02,48.50) | 35.56(32.47,38.66) |  |  |
| **Race ^b^** |  |  |  |  |  | 0.01 |  |
| Mexican American | 7.69(6.31, 9.08) | 7.02(4.84, 9.19) | 6.98(5.28, 8.67) | 7.60(5.72, 9.48) | 9.13(7.25,11.01) |  |  |
| Non-Hispanic Black | 9.55(7.65,11.46) | 10.83(8.32,13.33) | 8.47(6.58,10.35) | 8.61(6.39,10.83) | 10.54(7.42,13.66) |  |  |
| Non-Hispanic White | 71.51(62.32,80.70) | 70.02(65.64,74.40) | 72.01(67.75,76.26) | 74.66(70.55,78.77) | 69.25(62.89,75.60) |  |  |
| Other Hispanic | 4.79(3.53, 6.05) | 4.47(3.19,5.76) | 4.66(3.33,5.99) | 4.14(2.91,5.37) | 5.81(3.66,7.96) |  |  |
| Other Race- Including Multi-Racial | 6.45(5.14, 7.76) | 7.66(5.20,10.13) | 7.89(5.64,10.13) | 4.99(3.55, 6.43) | 5.27(2.88, 7.66) |  |  |
| **Education ^b^** |  |  |  |  |  | < 0.0001 |  |
| <University | 36.95(32.68,41.23) | 37.93(33.24,42.61) | 32.01(28.13,35.88) | 35.11(30.76,39.46) | 49.32(44.86,53.78) |  |  |
| >University | 59.00(53.06,64.93) | 62.07(57.39,66.76) | 67.99(64.12,71.87) | 64.89(60.54,69.24) | 50.68(46.22,55.14) |  |  |
| **Income ^b^** |  |  |  |  |  | 0.18 |  |
| <$20000 | 16.53(14.37,18.68) | 17.26(14.62,19.91) | 15.38(12.82,17.94) | 16.94(13.54,20.34) | 18.76(15.57,21.94) |  |  |
| >$20000 | 80.42(72.65,88.19) | 82.74(80.09,85.38) | 84.62(82.06,87.18) | 83.06(79.66,86.46) | 81.24(78.06,84.43) |  |  |
| **Smoke ^b^** |  |  |  |  |  | < 0.0001 |  |
| Non-smoker | 52.30(47.50,57.10) | 55.83(53.21,58.45) | 58.83(55.30,62.36) | 54.00(49.65,58.36) | 49.04(45.08,53.00) |  |  |
| Past-smoker | 23.53(21.28,25.78) | 25.06(22.43,27.70) | 26.23(23.63,28.82) | 26.16(22.42,29.89) | 20.62(17.94,23.31) |  |  |
| Current smoker | 20.16(17.86,22.46) | 19.11(16.73,21.49) | 14.94(12.34,17.55) | 19.84(17.32,22.35) | 30.33(27.14,33.53) |  |  |
| **Thyroid ^b^** |  |  |  |  |  | 0.16 |  |
| Normal | 93.81(86.37,101.26) | 94.24(92.55,95.93) | 92.76(91.03,94.49) | 94.72(93.10,96.34) | 93.73(92.25,95.21) |  |  |
| Subclinical hypothyroidism | 2.05(1.50, 2.60) | 1.80(0.92,2.69) | 2.68(1.63,3.74) | 1.97(0.87,3.07) | 1.66(0.83,2.48) |  |  |
| Subclinical hyperthyroidism | 1.52(1.11, 1.92) | 1.91(0.95,2.86) | 1.43(0.67,2.20) | 1.51(0.69,2.33) | 1.28(0.63,1.94) |  |  |
| Overt hypothyroidism | 2.37(1.82 2.92) | 1.82(0.85,2.78) | 2.59(1.79,3.39) | 1.65(0.69,2.62) | 3.28(2.18,4.38) |  |  |
| Overt hyperthyroidism | 0.25(0.10, 0.40) | 0.23(-0.02,0.49) | 0.53(0.08,0.99) | 0.14(-0.02,0.31) | 0.05(-0.02,0.11) |  |  |
| AIT | 13.78(12.05,15.51) | 15.41(12.74,18.08) | 14.54(12.11,16.96) | 12.97(10.40,15.53) | 12.97(10.69,15.25) | 0.47 |  |

a: presented as mean ± SEM; b: presented as 95% confidence interval. P value less than 0.05 means statistically significant.

Table S4: Relationship between PAM and thyroid hormones in different populations after excluding thyroid antibody-positive participants.

| Total | Variable | Total | <840MET-  minutes/week | 840-2400MET-minutes/week | 2400-5960MET-minutes/week | >5960MET-  minutes/week | P value |
| --- | --- | --- | --- | --- | --- | --- | --- |
|  | Age | 44.29(43.38,45.21) | 47.02(45.76,48.29) | 46.08(45.17,46.99) | 43.79(42.09,45.49) | 40.84(39.65,42.03) | <0.0001 |
|  | BMI | 28.20(27.93,28.47) | 29.20(28.71,29.69) | 27.93(27.61,28.24) | 27.79(27.23,28.35) | 28.23(27.85,28.60) | < 0.001 |
|  | TSH | 1.77(1.72,1.81) | 1.93(1.76,2.10) | 1.77(1.68,1.86) | 1.76(1.69,1.82) | 1.66(1.59,1.72) | 0.01 |
|  | FT4 | 10.19(10.06,10.32) | 10.24(10.08,10.40) | 10.35(10.20,10.50) | 10.14(9.97,10.31) | 10.02(9.85,10.19) | 0.001 |
|  | FT3 | 3.20(3.18,3.22) | 3.17(3.14,3.20) | 3.14(3.11,3.17) | 3.23(3.19,3.26) | 3.27(3.25,3.30) | <0.0001 |
|  | TT4 | 7.74(7.65,7.82) | 7.93(7.77,8.09) | 7.80(7.71,7.89) | 7.68(7.55,7.81) | 7.59(7.48,7.70) | < 0.001 |
|  | TT3 | 114.27(112.84,  115.71) | 112.85(110.26,  115.44) | 112.05(110.38,  113.72) | 115.02(113.00,  117.03) | 117.15(115.16,  119.15) | < 0.001 |
| Men | Variable | Total | <1200MET-  minutes/week | 1200-3320MET-minutes/week | 3320-8400MET-minutes/week | >8400MET-minutes/week | P value |
|  | Age | 44.20(43.28,45.11) | 46.87(45.13,48.61) | 46.87(45.79,47.94) | 44.40(42.55,46.24) | 40.70(39.45,41.95) | <0.0001 |
|  | BMI | 28.36(28.06,28.65) | 29.19(28.35,30.02) | 28.41(27.97,28.86) | 28.07(27.64,28.51) | 28.19(27.73,28.65) | 0.10 |
|  | TSH | 1.78(1.71,1.84) | 1.93(1.76,2.09) | 1.80(1.63,1.96) | 1.82(1.72,1.92) | 1.66(1.59,1.74) | < 0.001 |
|  | FT4 | 10.19(10.05,10.34) | 10.22(10.00,10.44) | 10.44(10.28,10.60) | 10.19(10.00,10.38) | 9.98(9.80,10.16) | <0.0001 |
|  | FT3 | 3.30(3.27,3.32) | 3.28(3.23,3.32) | 3.24(3.21,3.28) | 3.30(3.26,3.34) | 3.35(3.32,3.38) | <0.0001 |
|  | TT4 | 7.52(7.45,7.60) | 7.69(7.49,7.89) | 7.59(7.49,7.70) | 7.51(7.38,7.64) | 7.41(7.30,7.52) | 0.02 |
|  | TT3 | 115.34(113.72,  116.95) | 114.14(111.34,  116.93) | 112.23(110.43,  114.02) | 115.75(113.07,  118.42) | 118.12(115.84,  120.40) | < 0.001 |
| Women | Variable | Total | <720MET-minutes/week | 720-1680MET-minutes/week | 1680-4000MET-minutes/week | >8804000MET-minutes/week | P value |
|  | Age | 44.41(43.26,45.56) | 47.13(45.80,48.45) | 45.34(43.99,46.69) | 42.94(40.77,45.12) | 41.11(39.27,42.95) | <0.0001 |
|  | BMI | 28.02(27.67,28.37) | 29.21(28.56,29.85) | 27.47(26.97,27.97) | 27.39(26.46,28.31) | 28.30(27.49,29.11) | 0.001 |
|  | TSH | 1.75(1.67,1.83) | 1.93(1.65,2.22) | 1.75(1.67,1.82) | 1.67(1.56,1.78) | 1.65(1.56,1.73) | 0.08 |
|  | FT4 | 10.19(10.06,10.32) | 10.25(10.08,10.41) | 10.26(10.09,10.44) | 10.07(9.87,10.27) | 10.11(9.86,10.36) | 0.18 |
|  | FT3 | 3.08(3.06,3.11) | 3.09(3.06,3.13) | 3.04(3.01,3.07) | 3.12(3.08,3.16) | 3.11(3.07,3.15) | 0.00 |
|  | TT4 | 7.99(7.87,8.10) | 8.09(7.92,8.26) | 7.99(7.84,8.14) | 7.91(7.73,8.09) | 7.95(7.74,8.15) | 0.43 |
|  | TT3 | 113.00(111.35,  114.66) | 111.96(108.75,  115.17) | 111.88(109.54,  114.23) | 113.99(110.38,  117.61) | 115.18(111.79,  118.58) | 0.42 |

P value less than 0.05 means statistically significant.

Table S5: Relationship between PAT and thyroid hormones in different populations after excluding thyroid antibody-positive participants.

| Total | Variable | Total | <180 minutes/week | 180-480 minutes/week | 480-1200 minutes/week | >1200 minutes/week | P value |
| --- | --- | --- | --- | --- | --- | --- | --- |
|  | Age | 44.29(43.38,45.21) | 47.38(46.14,48.62) | 45.67(44.69,46.65) | 43.62(41.97,45.27) | 40.83(39.64,42.02) | <0.001 |
|  | BMI | 28.20(27.93,28.47) | 29.02(28.58,29.47) | 27.89(27.54,28.25) | 27.82(27.29,28.35) | 28.23(27.87,28.58) | < 0.001 |
|  | TSH | 1.77(1.72,1.81) | 1.90(1.75,2.06) | 1.77(1.67,1.87) | 1.74(1.68,1.81) | 1.66(1.60,1.73) | 0.01 |
|  | FT4 | 10.19(10.06,10.32) | 10.25(10.09,10.40) | 10.36(10.20,10.51) | 10.12(9.95,10.29) | 10.04(9.86,10.21) | 0.001 |
|  | FT3 | 3.20(3.18,3.22) | 3.16(3.13,3.18) | 3.14(3.11,3.17) | 3.23(3.20,3.26) | 3.27(3.25,3.30) | <0.0001 |
|  | TT4 | 7.74(7.65,7.82) | 7.92(7.77,8.07) | 7.78(7.69,7.88) | 7.66(7.54,7.78) | 7.60(7.49,7.71) | < 0.001 |
|  | TT3 | 114.27(112.84,  115.71) | 112.75(110.45,  115.04) | 112.02(110.14,  113.91) | 115.08(113.08,  117.08) | 117.23(115.15,  119.30) | < 0.001 |
| Men | Variable | Total | <240 minutes/week | 240-633 minutes/week | 633-1520 minutes/week | >1520 minutes/week | P value |
|  | Age | 44.20(43.28,45.11) | 47.38(45.63,49.12) | 46.54(45.38,47.70) | 44.11(42.35,45.88) | 40.71(39.48,41.95) | <0.001 |
|  | BMI | 28.36(28.06,28.65) | 29.39(28.57,30.20) | 28.20(27.76,28.65) | 28.09(27.68,28.51) | 28.18(27.73,28.64) | 0.02 |
|  | TSH | 1.78(1.71,1.84) | 1.89(1.74,2.03) | 1.81(1.63,1.99) | 1.80(1.71,1.89) | 1.67(1.59,1.75) | 0.001 |
|  | FT4 | 10.19(10.05,10.34) | 10.26(10.02,10.49) | 10.44(10.26,10.62) | 10.15(9.96,10.33) | 10.00(9.82,10.19) | <0.001 |
|  | FT3 | 3.30(3.27,3.32) | 3.26(3.22,3.30) | 3.25(3.21,3.29) | 3.30(3.26,3.35) | 3.35(3.32,3.38) | < 0.001 |
|  | TT4 | 7.52(7.45,7.60) | 7.67(7.47,7.87) | 7.59(7.48,7.70) | 7.49(7.36,7.62) | 7.42(7.31,7.54) | 0.03 |
|  | TT3 | 115.34(113.72,  116.95) | 113.84(111.27,  116.41) | 112.22(110.24,  114.20) | 115.90(113.39,  118.41) | 118.13(115.76,  120.51) | 0.001 |
| Women | Variable | Total | <150 minutes/week | 150-360 minutes/week | 360-880 minutes/week | >880 minutes/week | P value |
|  | Age | 44.41(43.26,45.56) | 47.38(46.19,48.58) | 44.81(43.29,46.33) | 42.90(40.80,44.99) | 41.06(39.15,42.98) | < 0.001 |
|  | BMI | 28.02(27.67,28.37) | 28.78(28.22,29.33) | 27.58(27.07,28.10) | 27.42(26.50,28.34) | 28.31(27.54,29.09) | 0.01 |
|  | TSH | 1.75(1.67,1.83) | 1.91(1.67,2.16) | 1.73(1.65,1.82) | 1.66(1.56,1.77) | 1.65(1.56,1.74) | 0.10 |
|  | FT4 | 10.19(10.06,10.32) | 10.24(10.09,10.40) | 10.27(10.09,10.45) | 10.08(9.89,10.28) | 10.10(9.83,10.37) | 0.23 |
|  | FT3 | 3.08(3.06,3.11) | 3.08(3.05,3.11) | 3.04(3.01,3.08) | 3.12(3.08,3.17) | 3.11(3.07,3.16) | 0.001 |
|  | TT4 | 7.99(7.87,8.10) | 8.09(7.92,8.26) | 7.97(7.82,8.12) | 7.91(7.72,8.09) | 7.96(7.74,8.17) | 0.38 |
|  | TT3 | 113.00(111.35,  114.66) | 112.00(109.11,  114.89) | 111.83(109.06,  114.60) | 113.88(110.23,  117.54) | 115.39(111.79,  118.99) | 0.41 |

P value less than 0.05 means statistically significant.

Table S6: Correlation between PA and FT4, TSH in men: Pearson and Spearman correlation coefficient.

|  | Variable | FT4 | TSH | PAT | PAM |
| --- | --- | --- | --- | --- | --- |
|  | FT4 | 1 | -0.135295915 | -0.066157458 | -0.060571304 |
| Pearson | TSH | -0.135295915 | 1 | -0.056136373 | -0.056461203 |
|  | PAT | -0.066157458 | -0.056136373 | 1 | 0.970913422 |
|  | PAM | -0.060571304 | -0.056461203 | 0.970913422 | 1 |
|  | Variable | FT4 | TSH | PAT | PAM |
|  | FT4 | 1 | -0.076269976 | -0.068317286 | -0.064932001 |
| Spearman | TSH | -0.076269976 | 1 | -0.082740479 | -0.090977562 |
|  | PAT | -0.068317286 | -0.082740479 | 1 | 0.983468581 |
|  | PAM | -0.064932001 | -0.090977562 | 0.983468581 | 1 |

Table S7: Multiple tests of physical activity and thyroid function disorders: the Tukey-Kramer corrections.

|  | PAT | Total | | Men | | PAM | Total | | Men | |
| --- | --- | --- | --- | --- | --- | --- | --- | --- | --- | --- |
|  |  | Estimate | P value | Estimate | P value |  | Estimate | P value | Estimate | P value |
| SHYPO | Q2 -Q1 | 0.3032 | 0.614 | -0.00229 | 1 | Q2 -Q1 | 0.19356 | 0.87087 | 0.09192 | 0.9946 |
|  | Q3 -Q1 | -0.1077 | 0.978 | 0.233965 | 0.9105 | Q3 -Q1 | 0.14665 | 0.93968 | 0.06522 | 0.998 |
|  | Q4 -Q1 | -0.6043 | 0.222 | -1.51207 | 0.0756 | Q4 -Q1 | -0.96541 | 0.03298 | -1.52394 | 0.0749 |
|  | Q3 -Q2 | -0.4109 | 0.392 | 0.236256 | 0.9223 | Q3 -Q2 | -0.04692 | 0.99752 | -0.0267 | 0.9999 |
|  | Q4 -Q2 | -0.9075 | 0.018 | -1.50978 | 0.0858 | Q4 -Q2 | -1.15897 | 0.00485 | -1.61586 | 0.0499 |
|  | Q4 -Q3 | -0.4966 | 0.421 | -1.74604 | 0.0265 | Q4 -Q3 | -1.11206 | 0.0077 | -1.58916 | 0.0555 |
| OHYPO |  |  |  |  |  |  |  |  |  |  |
|  | Q2 -Q1 | 0.26601 | 0.685 | -0.57851 | 0.432 | Q2 -Q1 | 0.20234 | 0.833 | -0.58763 | 0.405 |
|  | Q3 -Q1 | 0.04108 | 0.998 | -0.09864 | 0.99 | Q3 -Q1 | 0.01478 | 1 | -0.05762 | 0.998 |
|  | Q4 -Q1 | 0.37429 | 0.372 | 0.11361 | 0.982 | Q4 -Q1 | 0.26341 | 0.678 | 0.12886 | 0.976 |
|  | Q3 -Q2 | -0.22493 | 0.79 | 0.47987 | 0.62 | Q3 -Q2 | -0.18756 | 0.862 | 0.53001 | 0.506 |
|  | Q4 -Q2 | 0.10828 | 0.964 | 0.69211 | 0.272 | Q4 -Q2 | 0.06107 | 0.993 | 0.71649 | 0.217 |
|  | Q4 -Q3 | 0.33321 | 0.485 | 0.21225 | 0.911 | Q4 -Q3 | 0.24863 | 0.716 | 0.18648 | 0.935 |
| SHYPER |  |  |  |  |  |  |  |  |  |  |
|  | Q2 -Q1 | 0.02728 | 1 | 0.4491 | 0.782 | Q2 -Q1 | -0.12585 | 0.97 | 0.566843 | 0.634 |
|  | Q3 -Q1 | -0.2095 | 0.883 | 0.2244 | 0.968 | Q3 -Q1 | -0.23047 | 0.853 | -0.16038 | 0.992 |
|  | Q4 -Q1 | -0.2972 | 0.749 | 0.3374 | 0.894 | Q4 -Q1 | -0.27632 | 0.778 | 0.575872 | 0.622 |
|  | Q3 -Q2 | -0.23678 | 0.852 | -0.2247 | 0.962 | Q3 -Q2 | -0.10463 | 0.985 | -0.72722 | 0.467 |
|  | Q4 -Q2 | -0.32448 | 0.715 | -0.1118 | 0.995 | Q4 -Q2 | -0.15048 | 0.959 | 0.009029 | 1 |
|  | Q4 -Q3 | -0.0877 | 0.992 | 0.113 | 0.995 | Q4 -Q3 | -0.04585 | 0.999 | 0.736248 | 0.456 |

SHYPO: Subclinical hypothyroidism; OHYPO: Overt hypothyroidism; SHYPER: Subclinical hyperthyroidism.

Table S8: Multiple tests of physical activity and TSHL, TSHH, FT4L, FT4H: the Tukey-Kramer corrections.

|  | PAT | Total | | Men | | PAM | Total | | Men | |
| --- | --- | --- | --- | --- | --- | --- | --- | --- | --- | --- |
|  |  | Estimate | P value | Estimate | P value |  | Estimate | P value | Estimate | P value |
| TSHL | Q2 - Q1 | -0.00768 | 1 | 0.195944 | 0.33835 | Q2 - Q1 | -0.03274 | 0.9797 | 0.132 | 0.67736 |
|  | Q3 - Q1 | 0.095269 | 0.634 | 0.202416 | 0.2821 | Q3 - Q1 | 0.09051 | 0.6884 | 0.21483 | 0.24917 |
|  | Q4 - Q1 | 0.182363 | 0.107 | 0.361152 | 0.00676 | Q4 - Q1 | 0.19007 | 0.0895 | 0.41813 | 0.00155 |
|  | Q3 - Q2 | 0.102949 | 0.61 | 0.006472 | 0.99994 | Q3 - Q2 | 0.12325 | 0.4481 | 0.08283 | 0.88795 |
|  | Q4 - Q2 | 0.190044 | 0.11 | 0.165208 | 0.4799 | Q4 - Q2 | 0.22281 | 0.0342 | 0.28612 | 0.05576 |
|  | Q4 - Q3 | 0.087095 | 0.706 | 0.158737 | 0.48722 | Q4 - Q3 | 0.09956 | 0.60565 | 0.2033 | 0.25982 |
| TSHH |  |  |  |  |  |  |  |  |  |  |
|  | Q2 - Q1 | -0.18073 | 0.1356 | -0.15387 | 0.564 | Q2 - Q1 | -0.19947 | 0.0808 | -0.11252 | 0.7659 |
|  | Q3 - Q1 | -0.21933 | 0.0367 | 0.000654 | 1 | Q3 - Q1 | -0.19464 | 0.0893 | -0.18879 | 0.3656 |
|  | Q4 - Q1 | -0.33622 | <0.001 | -0.35792 | 0.013 | Q4 - Q1 | -0.38136 | <0.006 | -0.34778 | 0.0202 |
|  | Q3 - Q2 | -0.0386 | 0.9711 | 0.154527 | 0.579 | Q3 - Q2 | 0.00483 | 0.9992 | -0.07627 | 0.9179 |
|  | Q4 - Q2 | -0.15549 | 0.3096 | -0.20405 | 0.378 | Q4 - Q2 | -0.18188 | 0.1656 | -0.23525 | 0.2195 |
|  | Q4 - Q3 | -0.1169 | 0.5469 | -0.35858 | 0.016 | Q4 - Q3 | -0.18671 | 0.144 | -0.15899 | 0.5673 |
| FT4L |  |  |  |  |  |  |  |  |  |  |
|  | Q2 - Q1 | -0.0585 | 0.942 | -0.23498 | 0.413 | Q2 - Q1 | 0.06195 | 0.93 | -0.17846 | 0.635 |
|  | Q3 - Q1 | -0.16283 | 0.383 | 0.03343 | 0.995 | Q3 - Q1 | -0.09288 | 0.812 | 0.05564 | 0.98 |
|  | Q4 - Q1 | 0.05444 | 0.948 | 0.06089 | 0.972 | Q4 - Q1 | 0.029 | 0.992 | 0.08664 | 0.931 |
|  | Q3 - Q2 | -0.10433 | 0.767 | 0.26841 | 0.308 | Q3 - Q2 | -0.15483 | 0.448 | 0.2341 | 0.394 |
|  | Q4 - Q2 | 0.11293 | 0.706 | 0.29587 | 0.223 | Q4 - Q2 | -0.03295 | 0.988 | 0.2651 | 0.285 |
|  | Q4 - Q3 | 0.21726 | 0.16 | 0.02746 | 0.997 | Q4 - Q3 | 0.12188 | 0.649 | 0.031 | 0.996 |
| FT4H |  |  |  |  |  |  |  |  |  |  |
|  | Q2 - Q1 | -0.00872 | 0.99955 | 0.04226 | 0.98048 | Q2 - Q1 | -0.02016 | 0.99461 | 0.04219 | 0.9806 |
|  | Q3 - Q1 | -0.10169 | 0.57522 | -0.21845 | 0.1897 | Q3 - Q1 | -0.04892 | 0.93088 | -0.12998 | 0.6453 |
|  | Q4 - Q1 | -0.28819 | 0.0028 | -0.33999 | 0.01251 | Q4 - Q1 | -0.27677 | 0.00494 | -0.30498 | 0.0392 |
|  | Q3 - Q2 | -0.09297 | 0.67695 | -0.2607 | 0.10565 | Q3 - Q2 | -0.02877 | 0.98509 | -0.17217 | 0.4079 |
|  | Q4 - Q2 | -0.27947 | 0.00671 | -0.38225 | 0.00579 | Q4 - Q2 | -0.25661 | 0.01229 | -0.34717 | 0.0132 |
|  | Q4 - Q3 | -0.1865 | 0.12493 | -0.12155 | 0.72596 | Q4 - Q3 | -0.22785 | 0.03417 | -0.175 | 0.433 |

Table S9: Association between PA and thyroid dysfunctions: p for trend.

|  | Thyroid dysfunctions | Total (p for trend) | Men (p for trend) | Women (p for trend) |
| --- | --- | --- | --- | --- |
| PAT | Overt hypothyroidism | 0.075 | 0.027 | 0.89 |
|  | Overt hyperthyroidism | 0.056 | 0.33 | 0.138 |
|  | Subclinical hypothyroidism | 0.034 | 0.025 | 0.543 |
|  | Subclinical hyperthyroidism | 0.289 | 0.043 | 0.023 |
| PAM |  |  |  |  |
|  | Overt hypothyroidism | 0.096 | 0.033 | 0.728 |
|  | Overt hyperthyroidism | 0.049 | 0.274 | 0.363 |
|  | Subclinical hypothyroidism | 0.201 | 0.02 | 0.728 |
|  | Subclinical hyperthyroidism | 0.174 | 0.07 | 0.008 |

Figure S1:


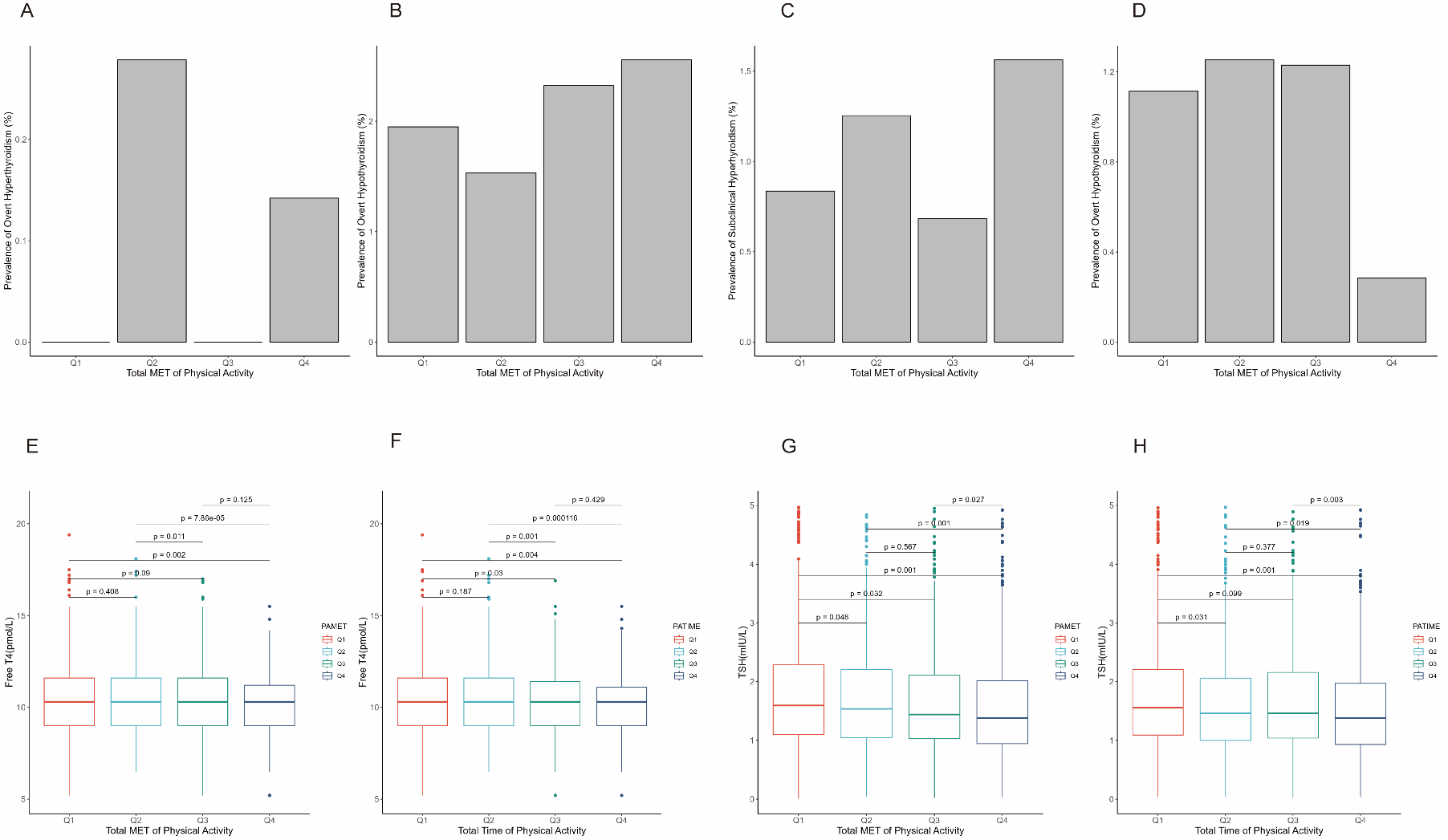


(A-D) Prevalence of thyroid disease in different intervals of PAM. A: Overt hyperthyroidism; B: Overt hypothyroidism; C: Subclinical hyperthyroidism; D: Subclinical hypothyroidism. (E-H) Bonferroni multiple corrections for FT4 and TSH in PAM and PAT.
